# Environmentally Induced Epigenetic Transgenerational Inheritance of Ovarian Disease

**DOI:** 10.1371/journal.pone.0036129

**Published:** 2012-05-03

**Authors:** Eric Nilsson, Ginger Larsen, Mohan Manikkam, Carlos Guerrero-Bosagna, Marina I. Savenkova, Michael K. Skinner

**Affiliations:** School of Biological Sciences, Center for Reproductive Biology, Washington State University, Pullman, Washington, United States of America; Massachusetts General Hospital, United States of America

## Abstract

The actions of environmental toxicants and relevant mixtures in promoting the epigenetic transgenerational inheritance of ovarian disease was investigated with the use of a fungicide, a pesticide mixture, a plastic mixture, dioxin and a hydrocarbon mixture. After transient exposure of an F0 gestating female rat during embryonic gonadal sex determination, the F1 and F3 generation progeny adult onset ovarian disease was assessed. Transgenerational disease phenotypes observed included an increase in cysts resembling human polycystic ovarian disease (PCO) and a decrease in the ovarian primordial follicle pool size resembling primary ovarian insufficiency (POI). The F3 generation granulosa cells were isolated and found to have a transgenerational effect on the transcriptome and epigenome (differential DNA methylation). Epigenetic biomarkers for environmental exposure and associated gene networks were identified. Epigenetic transgenerational inheritance of ovarian disease states was induced by all the different classes of environmental compounds, suggesting a role of environmental epigenetics in ovarian disease etiology.

## Introduction

Environmental exposures during fetal and early postnatal development can lead to an increased incidence of later life adult-onset diseases [1], [2], [3], [4]. Such environmental factors include nutritional abnormalities, stress and exposure to toxicants. Examples include fetal exposures to plasticizers such as bisphenol A leading to immune abnormalities [5], maternal smoking leading to increased pulmonary disease in adulthood [6], nutrition defects leading to hypertension in offspring [7], [8] and therapeutic drug exposure leading to vascular defects [9]. In addition to these direct effects of early life exposure on adult onset disease, environmental factors have been shown to affect the next F2 generation [8], [10], [11], [12]. The subsequent generations transgenerational inheritance of epigenetic changes in the genome now provides an additional molecular mechanism, along with classic induction of genetic mutations, for the germ line transmission of environmentally induced phenotypic change [2], [13], [14].

Effects on the F1 and F2 generation can be due to direct multigenerational exposure to the environmental factor [13], [15]. If a gestating female is defined as the F0 founder generation, then the fetal offspring are the F1 generation, and the germ cells present in those developing fetuses will eventually become the eggs or sperm that would form the F2 generation. An environmental exposure of an F0 generation gestating female directly exposes both the F1 generation fetuses and the germ cells present in those fetuses that will generate the F2 generation [2], [13], [15]. The subsequent F3 generation would be the first generation that would not have been directly exposed to the environmental factor. Therefore, effects on the F1 and F2 generation can be due to direct exposure and so should be considered multigenerational effects [13]. In contrast, a transgenerational effect following exposure of a F0 generation gestating female is defined as an effect seen in the F3 or later generations [15]. Transgenerational phenomena by definition do not involve direct exposure and have been shown to involve epigenetic changes induced in the germ line [16], [17], [18], [19].

The initial report of epigenetic transgenerational inheritance of adult onset disease was from gestating female rats exposed to the fungicide vinclozolin, in which F3 generation male offspring showed defects in sperm production [16], [20]. Transgenerational effects have also been reported after exposure of gestating rats to bisphenol A (BPA), where decreased fertility was seen in the F3 generation males [21]. Decreased fertility was also seen in F3 and F4 generation female mice after the gestating F0 generation was exposed to dioxin [22]. Similarly in mice, male F3 generation offspring showed changes in the methylation pattern of imprinted genes in sperm following exposure of the gestating F0 generation female to the agricultural fungicide vinclozolin [23]. Recently, a number of different exposures to environmental toxicants including BPA, phthalates, dioxin, pesticide, DEET and jet fuel hydrocarbons were found to promote epigenetic alterations in sperm and transgenerational inheritance of reproduction defects [19]. Therefore, a number of different environmental toxicants and other factors such as nutrition [4] can promote epigenetic transgenerational inheritance of adult onset disease.

In women, adult-onset diseases of the ovary that can dramatically affect fertility are primary ovarian insufficiency and polycystic ovarian disease. Primary ovarian insufficiency (POI) is characterized by a significant reduction in the primordial follicle pool of oocytes (eggs) that appears intrinsic to the ovary, and induction of menopause prior to age 40 [24]. This is associated with decreased estrogen and elevated gonadotropin levels in the blood. POI affects about 1% of women [25], [26], [27]. A reduced primordial follicle pool size correlated with POI has been shown in sheep and primates to also associate with polycystic ovarian disease [28], [29]. POI is often thought to have a genetic basis since chromosomal abnormalities and single gene mutations are associated with a percentage of POI cases. However, only a minority (4–20%) of human cases can be ascribed a genetic basis [26], [27], [30], [31], [32], [33], [34], [35], [36].

Polycystic ovarian (PCO) disease or polycystic ovary syndrome (PCOS) is a common endocrine disorder that affects 6–18% of women [37], [38], [39], [40], [41], [42]. It is characterized by infrequent ovulation or anovulation, high androgen levels in the blood, and the presence of multiple persistent ovarian cysts [43]. PCOS patients often show insulin resistance and a heightened risk for diabetes [44]. Current thought on the etiologies that lead to development of PCOS is that there are both genetic and environmental causal factors. A genetic predisposition in an individual may combine with an early-life environmental impact such as fetal stress or increased androgens *in utero* and lead to development of PCOS in adulthood [44], [45], [46]. Fetal or early postnatal exposure to androgens (e.g. di-hydrotestosterone) has been shown to promote PCO and associated clinical parameters (e.g. metabolic abnormalities, adiposity and endocrine abnormalities) in rats, mice and sheep [47], [48], [49]. Therefore, the rodent PCO model has many of the same clinical correlations that are seen with PCOS in humans. Sequence variations in several genes have been associated with PCOS [46], [50], [51], although at very low frequency and none are highly predictive. Epigenetic abnormalities such as those associated with non-random X-chromosome inactivation have also been linked to PCOS [51], [52], [53], [54]. Several diseases are now known to have an important epigenetic component, such as allergies [55], hepatic cancer [56], gastric cancer [57], asthma [58], colorectal cancer [59], prostate cancer [60], HIV latency [61] and psychiatric disorders [62]. The current study was designed to investigate the role of environmental epigenetics in ovarian disease.

A rat model is used to evaluate whether adult onset ovarian diseases are induced transgenerationally after exposure of a gestating F0 generation female to known environmental toxicants. The exposures are during days 8–15 of fetal development, which is the time of gonadal sex determination. The exposure compounds were: 1) Vinclozolin, an agricultural fungicide previously shown to cause transgenerational epigenetic disease [13], [20], [23]; 2) A mixture of permethrin, the most commonly used human insecticide shown to have minor toxicologic effects in mammals [63] and DEET, an insect repellent reported to have negligible toxic effects [63]; 3) A plastic mixture of bisphenol A (BPA), dibulylphthalate (DBP) and bis(2-ethylexyl)phthalate (DEHP), all plasticizer chemicals that commonly appear together from plastics with in vitro and in vivo toxic effects [64]; 4) Dioxin (TCDD), a by-product of some commercial chemical syntheses that has been shown to induce adult-onset diseases including premature acyclicity [65], [66]; and 5) Jet fuel (JP8), a hydrocarbon mixture (i.e. C3->C20) often used for dust control on road surfaces, with known toxicologic effects [67], [68], but is not known to induce reproductive defects [69]. The United States Department of Defense assisted in the selection of these toxicants and mixtures due to their relevance for exposures anticipated for military personnel. The plastic mixture included the three common toxicants present in heated bottled water, the pesticide mixture is the most common used in humans and the hydrocarbon mixture (i.e. jet fuel JP8) is commonly used in dust control on road surfaces. All of the above environmental toxicants have been implicated in inducing transgenerational disease phenotypes [13], [19], [21], [22]. The current study used pharmacological doses and administration to assess potential transgenerational actions on ovarian disease and should not be considered a risk assessment analysis. Future studies are now needed to do environmental risk assessment, based on the observations of the current studies.

The adult F3 generation females from each exposure lineage group were examined for the incidence of ovarian diseases similar to primary ovarian insufficiency and polycystic ovarian disease. The human ovarian diseases POI and PCOS have numerous other clinical conditions associated with them such as endocrine abnormalities and glucose intolerance. Therefore, the rat ovarian abnormalities/disease cannot be directly correlated to the clinical aspects of human ovarian disease, but do share the majority of morphological changes. In order to gain insight into possible cellular and molecular mechanisms involved in ovarian disease development, the granulosa cells from F3 generation vinclozolin and control lineage animals were evaluated for changes to their transcriptome and epigenome (DNA methylation pattern). All the primary cell types of an ovarian follicle such as the oocyte, theca cells and granulosa cells are anticipated to develop a transgenerationally altered transcriptome and epigenome [14], and so will participate in the adult onset disease development. The granulosa cell was selected to provide the proof of concept that such an alteration in genome activity could develop. Future studies will investigate the other cell types. The capacity of vinclozolin to directly induce oocyte loss in ovaries was also examined to clarify how F1 generation effects may develop. Observations demonstrate that the environmental toxicants examined induced transgenerational ovarian adult-onset disease, and suggest that primary ovarian insufficiency and polycystic ovarian disease can have an epigenetic transgenerational etiology.

## Results

### Transgenerational Ovarian Disease

Gestating female rats, designated as F0 generation animals, were treated by intraperitoneal injection daily from E8 (post conception gestational day 8) through E14. The F0 generation female rats received one of five different treatments as described in Methods: Vinclozolin, Pesticide (includes permethrin and DEET), Plastics (includes BPA, DBP and DEHP), Low-dose Plastics (50% of Plastics dose), Dioxin, Hydrocarbon (Jet fuel JP8), or DMSO vehicle as Control. The F1 generation offspring were bred to others of the same treatment group to produce an F2 generation, and F2 generation animals were similarly bred to produce an F3 generation (see Methods). No sibling or cousin breedings were used to avoid any inbreeding artifacts. Only the original F0 generation gestating female rats received the treatment exposures. Female rats from the F1 and F3 generations were kept until one year of age and then sacrificed. Ovaries were removed, fixed, sectioned and stained for histologic examination.

The number of oocytes (*i.e.* eggs) present in the ovaries was determined by counting follicles. Each ovarian follicle is composed of an oocyte surrounded by a layer of granulosa cells, a basement membrane and outer layers of thecal cells. Primordial follicles are in an arrested state of development and contain a single layer of squamous flattened granulosa cells. Developing follicles have multiple layers of proliferating granulosa cells and an increase in the oocyte diameter. Later in follicle development a fluid-filled antrum forms [70], [71], [72], [73]. In the F1 and F3 generation, ovarian morphological evaluation and counts were performed to determine the number of primordial follicles, pre-antral developing follicles and antral developing follicles as described in Methods [74].

**Figure 1:**
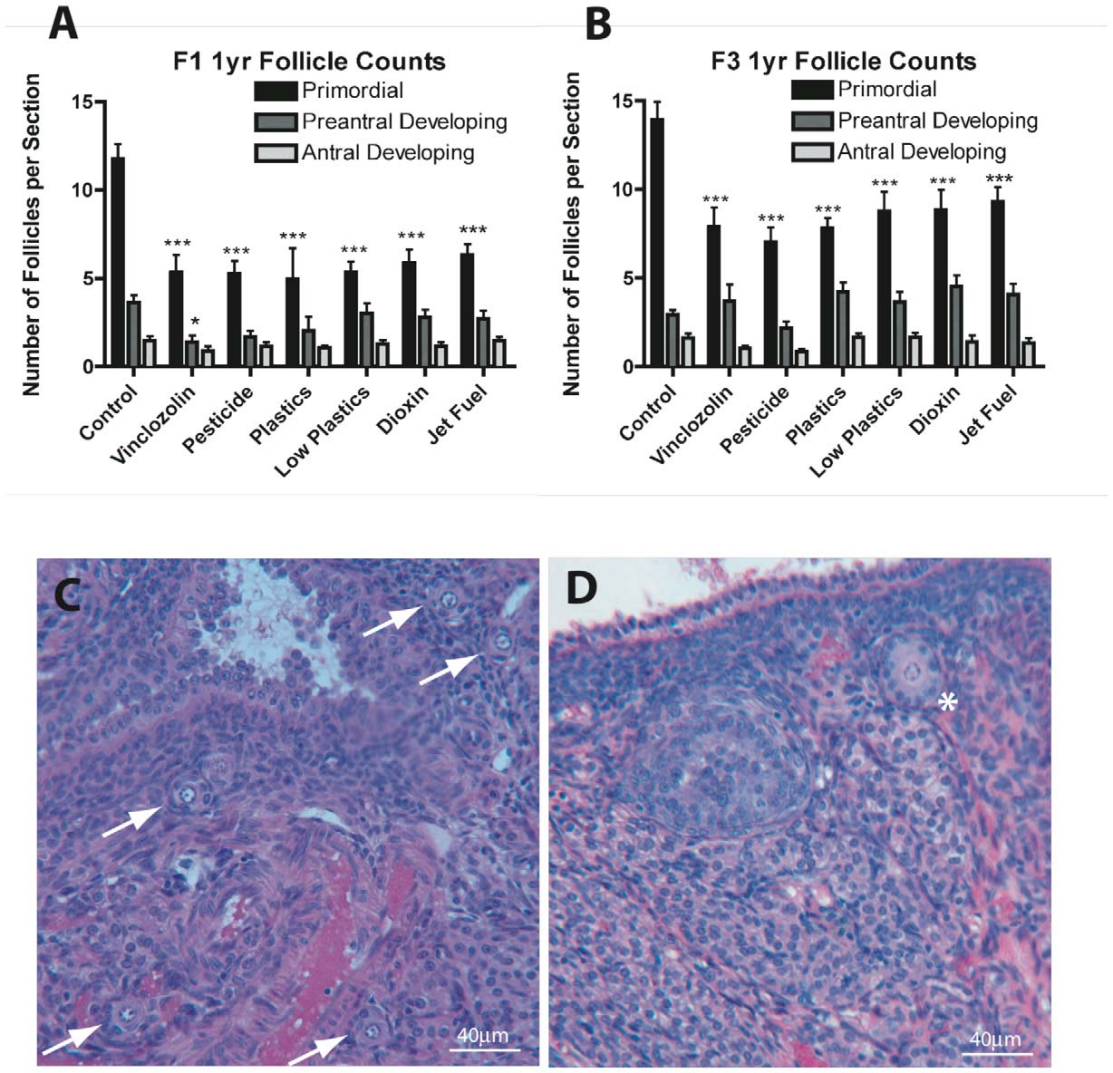
Follicle numbers and development. A) Number of primordial, preantral, and antral follicles per section in F1 generation ovaries. N = 9 animals per treatment group. B) Number of follicles per section in F3 generation ovaries. N = 9 animals per treatment group. Asterisks indicate groups significantly (*p≤0.05, ***p≤0.005) different than controls of their own follicle type by ANOVA followed by Dunnet's post-hoc test. C) H and E stained section of F3 generation control ovary showing several primordial follicles (arrows). D) H and E stained section of F3 generation vinclozolin lineage ovary without visible primordial follicles. Asterisk indicates a developing secondary follicle. Scale bar = 40 µm.

In F1 generation ovaries there was a marked and statistically significant (p<0.001) reduction in the number of primordial follicles in all exposure groups compared to ovaries from the vehicle-treated control lineage (Fig. 1). This indicates that the female fetuses exposed to these compounds during gonadal sex determination all have a decrease in their resting pool of primordial follicles. There was no change in the number of pre-antral developing follicles or antral developing follicles, except in the case of females of the vinclozolin-treated lineage. F1 vinclozolin group ovaries had significantly (p<0.05) fewer preantral developing follicles compared to controls (Fig. 1). This effect of the exposures on the F1 generation is attributed to direct fetal ovarian exposure to the treatments.

**Figure 2:**
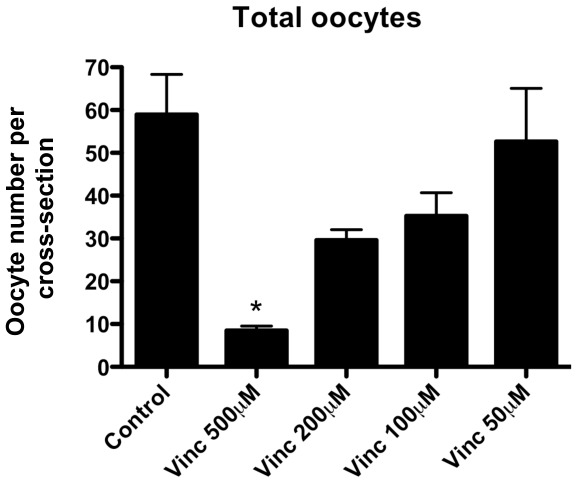
Number of oocytes per ovarian cross-section in ovaries taken from 4-day old rats and cultured whole for 10 days in the presence of different concentrations of vinclozolin. Data are from five different experiments performed in replicate. Asterisks indicate groups significantly (*p≤0.05) different than controls by ANOVA followed by Dunnet's post-hoc test.

Since treatment with environmental toxicants resulted in fewer oocytes being present in F1 generation adult ovaries compared to controls, an experiment was performed to assess if vinclozolin could act directly on ovaries to reduce oocyte number. Ovaries from four-day old rats, containing predominately primordial follicles, were placed into a whole-ovary culture system (see Methods) and treated *in vitro* for ten days with varying concentrations of vinclozolin. Treatment with 500 µM vinclozolin did result in a decrease (p<0.05) in oocyte number compared to controls (Fig. 2). The 200 µM and lower doses of vinclozolin did not significantly reduce oocyte number. Therefore, direct actions of vinclozolin on the F1 generation fetal gonad have the potential to reduce follicle numbers if the dose is sufficient.

In F3 generation ovaries, similarly to F1 females, there was a significant (p<0.001) reduction in the number of primordial follicles in all treatment groups, compared to controls (Fig. 1). Since none of these F3 generation animals were themselves exposed to the treatment compounds, this reduction in oocyte number is a transgenerational effect. There was no change in the number of preantral developing or antral developing follicles for any exposure lineage group compared to control lineage animals. Therefore, all the exposure groups examined induced a significant transgenerational decline in the primordial follicle pool size. For the purposes of this study, the ovaries of an animal were classified as having “disease” if the ovary primordial follicle numbers were ≥2 standard deviations less than that seen in controls. The incidence of the follicle pool disease was 33–60% across treatment groups.

**Figure 3:**
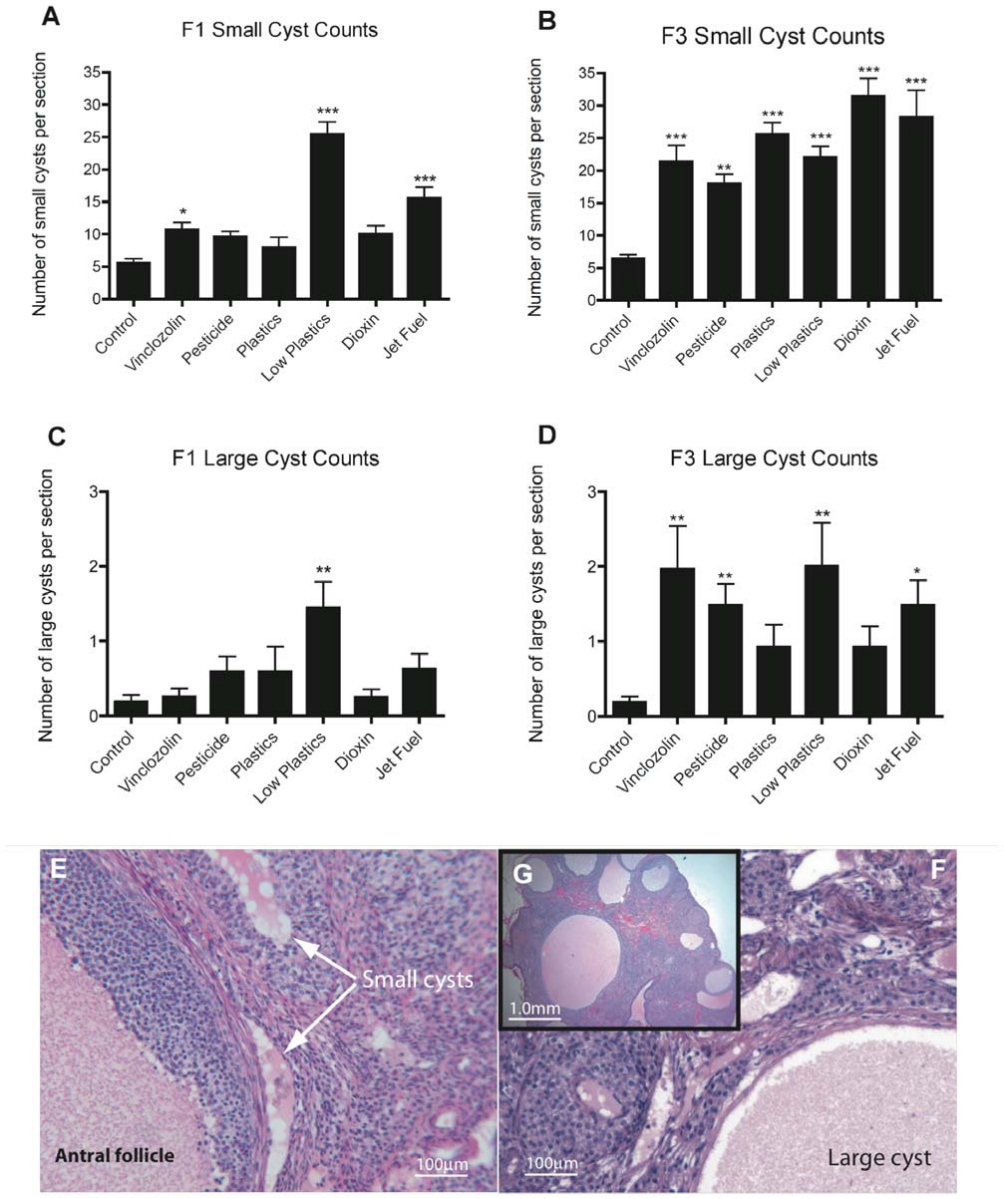
Ovarian cysts. Number of small (50–250 µm) cysts per section in F1 (A) and F3 (B) generation ovaries. Number of large (>250 µm) cysts per section in F1 (C) and F3 (D) generation ovaries. N = 9 animals per treatment group. Asterisks indicate groups significantly (*p≤0.05, **p≤0.01, ***p≤0.005) different than controls by ANOVA followed by Dunnet's post-hoc test. E) H and E stained section of F3 ovary showing small cysts. F) H and E stained F3 ovary showing a large cyst. G) Expanded view of small and large cysts.

Polycystic ovarian (PCO) disease is a common disease in humans, so ovaries from F1 and F3 generation animals were evaluated for the presence of cystic structures. Ovarian cysts were defined and categorized as either small or large cysts, as described in Methods. Interestingly, an increase in the number of both small and large cysts were seen most often in F3 generation ovaries from exposure lineages, rather than in F1 generation ovaries (Fig. 3). An increase (p<0.01) in small cysts was seen in all F3 generation treatment groups, compared to controls. However, in the F1 generation only the low-dose plastics, jet fuel hydrocarbons and vinclozolin lineage ovaries showed an increase (p<0.05) in small cysts. An increase in large cysts was observed in the F3 generation ovaries of the vinclozolin, pesticide, low-dose plastics and jet fuel treatment groups (Fig. 3). However, in F1 generation ovaries only the low-dose plastics showed an increase in the incidence of large cysts compared to controls. These results indicate that development of ovarian cysts occurs more often in the F3 generation, which demonstrates a transgenerational effect of the toxicant exposures. The large cysts observed in these ovaries often were lined with a sporadic single layer of epithelial granulosa cells and were surrounded by a band of theca cells (Fig. 3F). This is consistent with these large cysts being derived from antral follicles. However, some large cysts and associated cells were morphologically identified as being from *corpora lutea* [19]. These luteal cysts were present frequently in the F3 generation jet fuel hydrocarbon exposure lineage ovaries, Figure 3.

**Figure 4:**
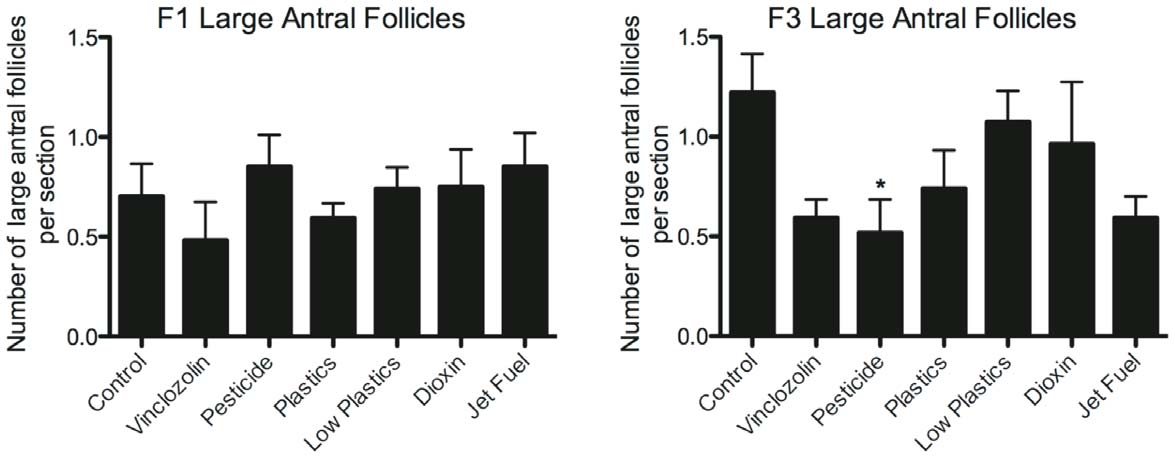
Large antral follicles. A) Number of large antral follicles per section in F1 generation ovaries. B) Number of large antral follicles per section in F3 generation ovaries. N = 9 animals per treatment group. Asterisks indicate groups significantly (*p≤0.05) different than controls by ANOVA followed by Dunnet's post-hoc test.

The number of healthy-looking large antral follicles was not found to be different between exposure and control groups in either the F1 or F3 generation ovaries (Fig. 4A & B). The exception was that there were significantly fewer (p<0.05) large antral follicles in the F3 pesticide-lineage ovaries. Therefore, the antral follicle development process appears relatively normal in the F1 and F3 generation females independent of exposure lineage.

Previous studies have demonstrated that negligible endocrine abnormalities are detected in 120-day-old F3 generation female rats following exposure to any of the toxicants studied [19], [75]. PCO has previously been associated with an increase in androgen serum levels which is due to the highly steroidogenic theca cells of the cysts. Theca cells primarily produce androstenedione so the serum androstenedione levels in the F3 generation 1-year-old females were examined. Preliminary studies show that the F3 generation control lineage had 47±3 pg/mL and the vinclozolin lineage had 177±82 pg/mL serum androstenedione. Therefore, the androgen levels were increased in the F3 generation vinclozolin lineage females that had the PCO disease. This increase in androgen levels requires further investigation as do the other associated clinical conditions of glucose intolerance, abnormal adiposity and hyperinsulemia.

### Transgenerational Granulosa Cell Transcriptome

**Figure 5:**
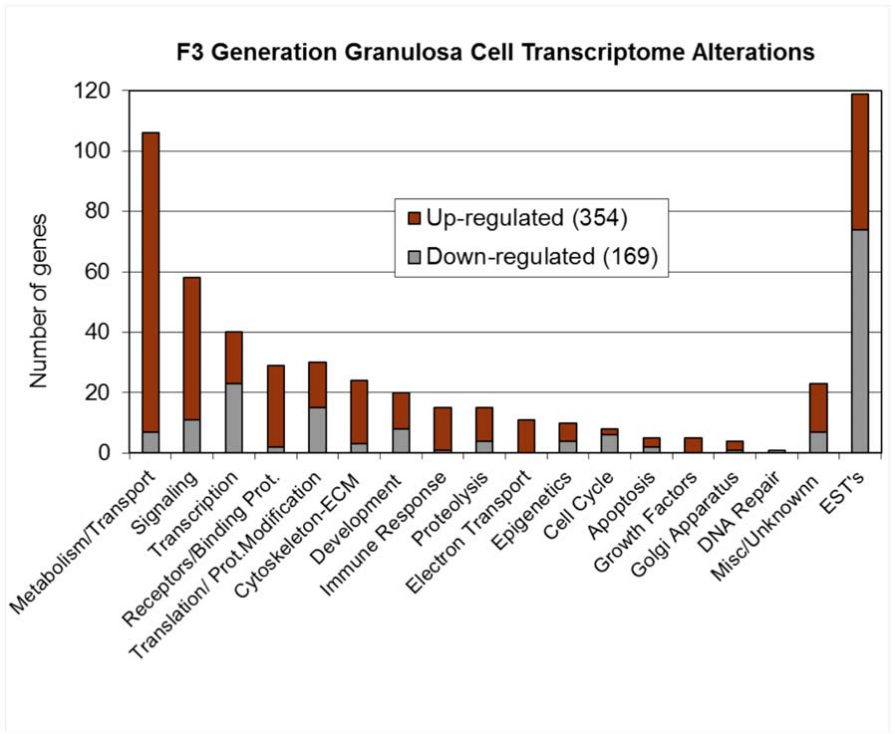
Number of genes with mRNA expression levels significantly different between Control and Vinclozolin-lineage F3 generation granulosa cells. Genes are placed into functional categories.

Previous studies demonstrated the vinclozolin induced epigenetic transgenerational inheritance of adult onset disease involving epigenetic modifications of the sperm [16] and heritable phenotypes through the paternal lineage [20]. The only cell that can transmit an altered epigenome between generations is the germline [13], however, all the cells derived from this sperm will have an altered epigenome transcriptome [14]. In order to see if transgenerational changes in gene expression are apparent in ovarian follicle cells of the exposure lineage females, the transcriptomes of granulosa cells from control and vinclozolin lineage ovaries were compared. Granulosa cells were collected from pre-ovulatory follicles of five-month old F3 generation vinclozolin and control lineage ovaries as described in Methods. Messenger RNA was isolated from the granulosa cells of each animal (n = 24) and RNA from four animals of the same treatment group were pooled to create three different pooled samples from each of the two treatment groups. Three F3 generation vinclozolin-lineage and three control-lineage mRNA pooled samples were used in a microarray analysis as described in Methods to evaluate alterations in gene expression. The analysis demonstrated that 523 genes were differentially expressed between control and vinclozolin lineage F3 generation granulosa cells (Table S1). The number of differentially expressed genes in each of several functional gene categories is shown in Figure 5 with the number of up-regulated and down-regulated genes indicated. Many of the differentially expressed genes were identified as contributing to metabolism or signaling processes. The complete list of differentially expressed genes is functionally categorized and presented in Table S1.

**Figure 6:**
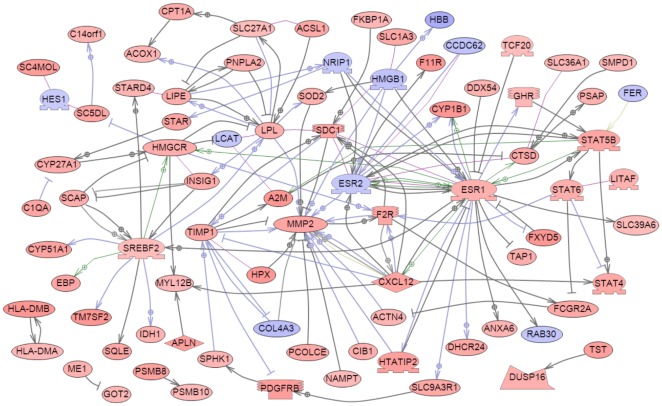
Gene network of known relationships among those genes found to be differentially expressed in Control compared to Vinclozolin-lineage F3 generation granulosa cells. Network is derived from an un-biased search of literature using Pathway Studio™. Node shapes code: oval and circle – protein; diamond – ligand; irregular polygon – phosphatase; circle/oval on tripod platform – transcription factor; ice cream cone – receptor. Red color represents up-regulated genes, blue color – down-regulated genes, grey rectangles represent cell processes; arrows with plus sign show positive regulation/activation, arrows with minus sign – negative regulation/inhibition. Grey arrows represent regulation, lilac – expression, green – promoter binding, olive – protein modification.

**Figure 7:**
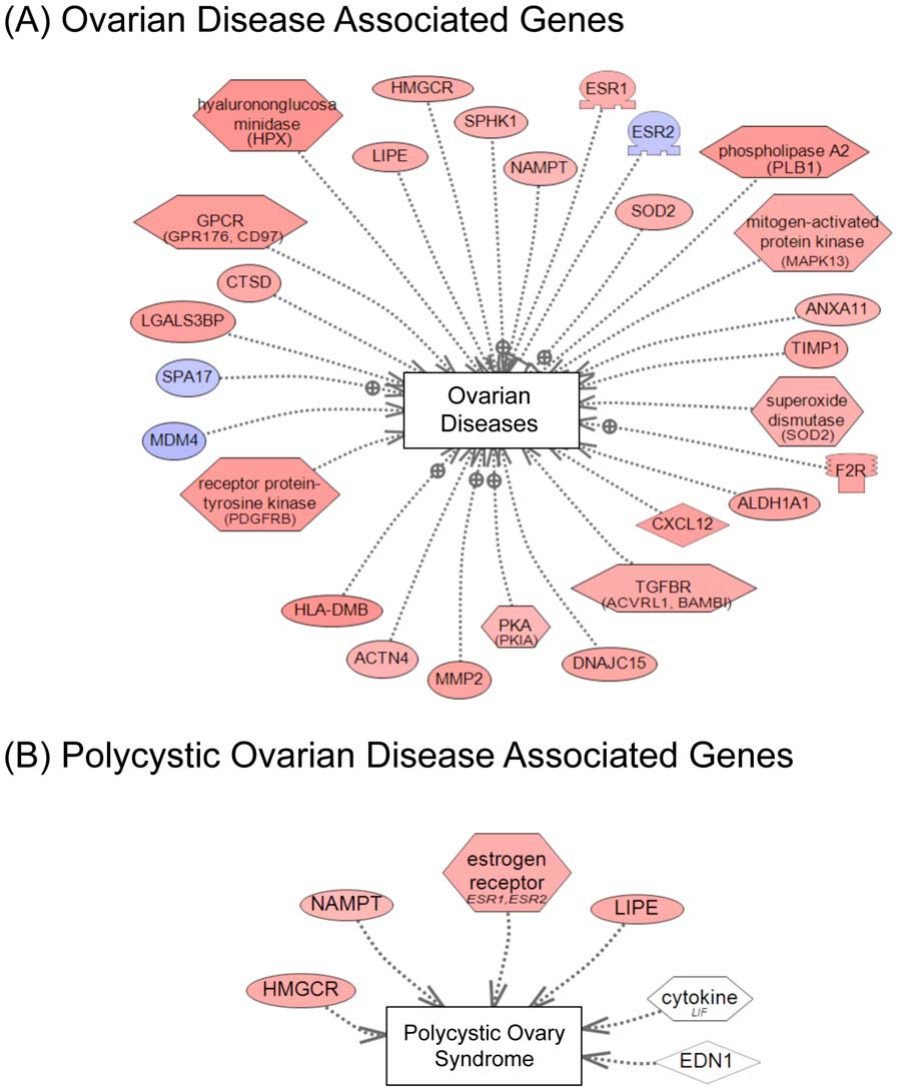
Ovarian diseases associated differentially expressed genes between F3 generation vinclozolin and control lineage granulosa cell. Sub-networks were identified using global literature analysis with Pathway Studio™. Node and arrow code is the same as for Figure 6. **A:** General ovarian diseases associated genes. **B:** Polycystic ovarian disease associated genes. White color nodes indicate differential methylated regions identified in this study.

A table of cellular pathways and processes impacted by the genes differentially expressed in vinclozolin lineage F3 generation granulosa cells is presented (Table 1). In Figure S2, two of the more heavily impacted cellular pathways are shown, PPAR signaling and steroid biosynthesis, with the differentially expressed genes highlighted. These data indicate that gene expression is altered transgenerationally in granulosa cells and that specific physiological processes may be affected by these changes. Additional bioinformatic analyses examined the functional relationships among the F3 generation differentially expressed genes identified. An unbiased literature based network analysis was performed as described in Methods to determine which genes are functionally linked with respect to binding, signaling or regulation. This created a gene network of direct connections as shown in Figure 6. Some genes show significant functional connections to others, such as ESR1, MMP2 and CXCL12. Such highly connected genes may play important regulatory roles in these F3 generation granulosa cells and in the development of ovarian disease states. Therefore, a transgenerational change in the granulosa cell transcriptome was identified that may be in part a causal factor in the molecular etiology of the transgenerational ovarian disease. Further analysis of the 523 genes with transgenerational alterations in gene expression identified previously known genes involved in ovarian disease and more specifically polycystic ovarian disease. A total of 30 genes were found to be related to ovarian disease and 5 directly related to PCO disease, Figure 7. Therefore, genes known to have a relationship with PCO and ovarian disease were shown to have altered expression. The potential role of the transgenerational change in the granulosa cell epigenome to promote this transcriptome alteration is described below.

**Table 1:** Physiological Pathway Enrichment.

Pathway Name	# Input Genes in Pathway	Impact Factor**
PPAR signaling pathway	11	17.0
Phagosome	11	
Cell adhesion molecules (CAMs)	10	10.3
Endocytosis	10	
Steroid biosynthesis	9	
Peroxisome	8	
Antigen processing and presentation	8	38.5
Leukocyte transendothelial migration	8	8.9
Fatty acid metabolism	6	
Valine, leucine and isoleucine degradation	6	
Spliceosome	6	
Lysosome	6	
Fc gamma R-mediated phagocytosis	6	
Regulation of actin cytoskeleton	6	4.7
Cysteine and methionine metabolism	5	
Glutathione metabolism	5	
Glycerophospholipid metabolism	5	
Biosynthesis of unsaturated fatty acids	5	10.2
MAPK signaling pathway	5	2.9
Neuroactive ligand-receptor interaction	5	1.5
Focal adhesion	5	5.0
Purine metabolism	4	
Lysine degradation	4	
Phenylalanine metabolism	4	
Sphingolipid metabolism	4	
Base excision repair	4	7.7
Calcium signaling pathway	4	4.4
Adherens junction	4	7.7
Tight junction	4	5.7
Complement and coagulation cascades	4	7.2
Jak-STAT signaling pathway	4	3.8
Pathways in cancer	4	2.3

Using 523 differentially expressed genes (F3 vinclozolin *vs.* control granulosa) in KEGG pathway database.

** Calculated by Pathway Express to estimate importance of these genes to pathway.

### Transgenerational Granulosa Cell Epigenome

**Figure 8:**
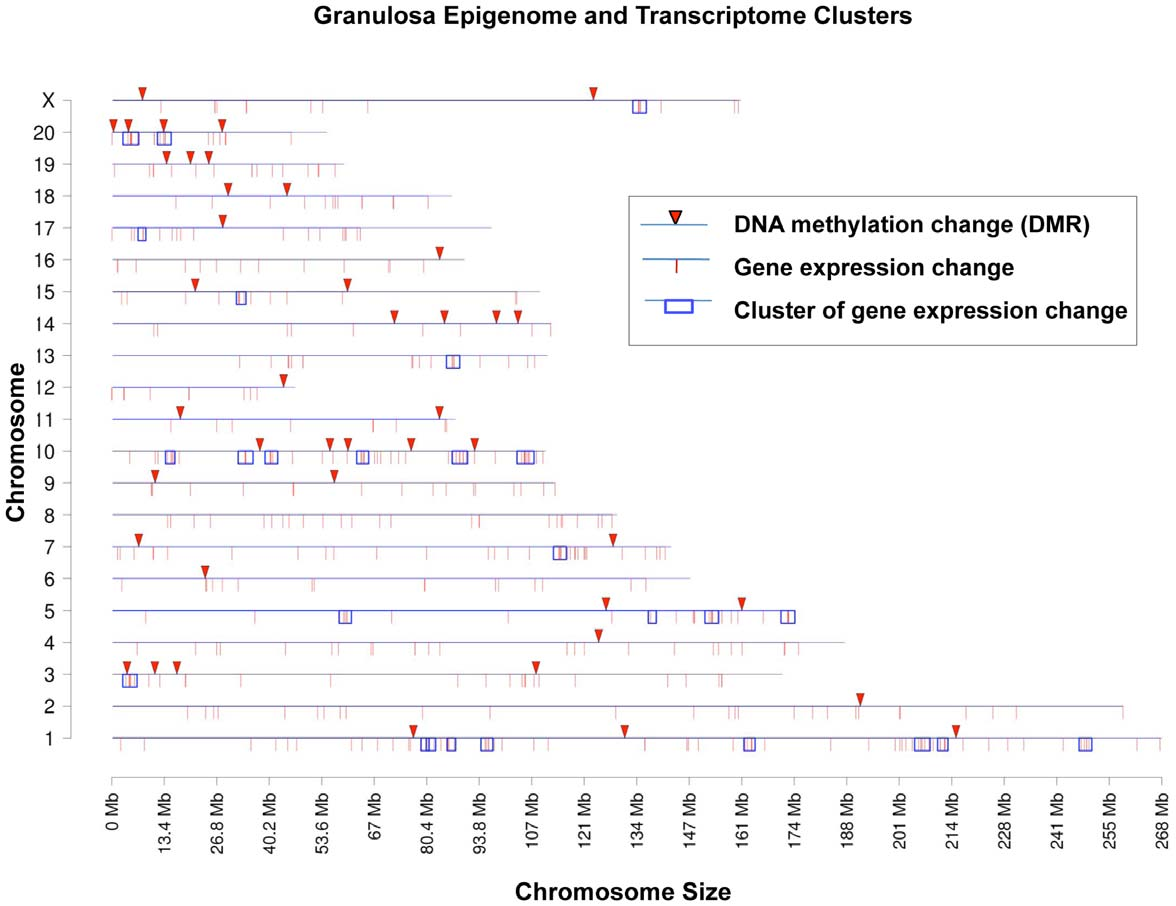
Chromosomal plot showing regions with vinclozolin-induced transgenerational changes in granulosa cells. Differential DNA methylation regions are displayed as inverted red triangles, changes in gene expression are displayed as red ticks, and a significant gene cluster of these genes with changed expression is delineated with blue open boxes. The chromosome number on Y-axis and size on X-axis are presented.

As previously described [13], [16], [20], an epigenetic transgenerational alteration of the sperm in vinclozolin lineage F3 generation animals can promote a transgenerational change in the epigenome unique to each cell type in all somatic cells derived from this germ line [14], [76]. The F3 generation vinclozolin lineage alterations in differentially DNA methylated regions (DMR) in the granulosa cells was investigated. For this, a methylated DNA immunoprecipitation (MeDIP) procedure was used, followed by comparative hybridization on a genome wide promoter tiling array (Chip), termed an MeDIP-Chip assay, as previously described [16]. The MeDIP-Chip analysis of the differential DNA methylation between control and vinclozolin lineage F3 generation granulosa cells identified 43 DMR with a statistical significance p>10^−7^, Table 2. The chromosomal locations of all the DMR are presented in Figure 8 and indicates most autosomes are involved. A comparison of the 43 DMR identified with the 523 differentially expressed granulosa cell genes demonstrated only 1 gene promoter with overlap (*Plekhm1*). Analysis of the probability for a random overlap between the 43 DMR and the 523 differentially expressed genes indicated that an overlap of 1.47 genes would be expected. Therefore, the one gene overlap is likely not significant. The vast majority of differentially expressed genes did not have a DMR present in their promoters. Further analysis using statistically significant over represented clusters of differentially expressed genes identified 26 clusters from 2–5 Mbase size that had 4 to 9 genes each, Table S2. An overlap of these regulated gene clusters with the DMR identified 3 overlapped clusters, Figure 8. These 2–5 Mbase regions we refer to as potential Epigenetic Control Regions (ECR). The hypothesis is that the epigenetic regulatory site (e.g. DMR) regulates distally the expression of genes within this ECR. This is likely mediated through non-coding RNA, similar to what is seen for imprinting control regions (ICR) previously identified [77]. A limited number of long non-coding RNA in the rat have been characterized, but of the 20 characterized 3 (NEAT1(chr1:204.8), khps1a (chr10:103.4), Zfhx2as (chr15:31.8)) had an overlap with the ECR identified. Further analysis of the rat lncRNA is needed before future correlation with the ECR can be made. The ECR provide one explanation for how a limited number of DMR can potentially control a large number of differentially regulated genes. The locations of the potential ECR are included in Figure 8 to correlate with the DMR identified.

**Table 2:** Differential DNA methylation regions (DMR) in F3 generation granulosa cells.

				Changed region coordinates			
Gene symbol	Gene Description	Entrez gene ID	Significance (p≤)	Chr	Start	End	Region size (bp)
Ceacam9	Carcinoembryonic antigen-related cell adhesion molecule 9	116711	8.53E-46	1	76960699	76961299	600
Sv2b	Synaptic vesicle glycoprotein 2b	117556	1.93E-61	1	130887128	130887728	600
Dtx4	Deltex homolog 4 (Drosophila)	293774	4.98E-14	1	215416136	215416736	600
Vdac1	Voltage-dependent anion channel 1	83529	2.12E-11	10	37793541	37794141	600
Rpl26	Ribosomal protein L26	287417	1.75E-28	10	55660989	55661674	685
Olr1468	olfactory receptor 1468	404977	1.23E-13	10	60268062	60268662	600
Cuedc1	CUE domain containing 1	303419	1.28E-08	10	76394145	76394745	600
Plekhm1	pleckstrin homology domain containing, family M (with RUN domain) member 1	303584	1.17E-34	10	92604304	92604904	600
RGD1563888	similar to DNA segment, Chr 16, ERATO Doi 472, expressed	360692	1.71E-31	11	17477532	17478132	600
Olr1567	olfactory receptor 1567	287970	2.23E-29	11	83619053	83619653	600
Selplg	selectin P ligand	363930	8.29E-10	12	43842412	43843012	600
Prom1	prominin 1	60357	3.57E-08	14	72118855	72119731	876
Lif	leukemia inhibitory factor	60584	5.55E-08	14	84886415	84887207	792
C1d	C1D nuclear receptor co-repressor	289810	1.46E-08	14	98152531	98153207	676
Zrsr1	zinc finger (CCCH type), RNA binding motif and serine/arginine rich 1	498425	1.69E-10	14	103657219	103657819	600
Gnpnat1	glucosamine-phosphate N-acetyltransferase 1	498486	8.05E-104	15	21287426	21288462	1036
Hars	histidyl-tRNA synthetase	307492	2.25E-08	15	60151437	60152140	703
LOC689713	LRRGT00175	689713	5.22E-30	16	83676467	83677258	791
Edn1	endothelin 1	24323	1.12E-11	17	28311735	28312730	995
Pcdha5	protocadherin alpha 5	393087	1.40E-39	18	29691331	29692216	885
Dtwd2	DTW domain containing 2	361326	3.36E-18	18	44718539	44719362	823
Mcm5	minichromosome maintenance complex component 5	291885	6.05E-15	19	13975188	13975788	600
Adcy7	adenylate cyclase 7	84420	4.10E-08	19	20076391	20076991	600
Dhps	deoxyhypusine synthase	288923	7.38E-10	19	24744799	24745688	889
Sv2a	synaptic vesicle glycoprotein 2a	117559	8.57E-27	2	190988570	190989259	689
Olr1686	olfactory receptor gene	294152	2.06E-09	20	417292	417892	600
Agpat1	1-acylglycerol-3-phosphate O-acyltransferase 1 (lysophosphatidic acid acyltransferase, alpha)	406165	1.91E-15	20	4243966	4244790	824
LOC686922	glutathione S-transferase, theta 4	686922	1.22E-09	20	13236150	13237231	1081
Unc5b	unc-5 homolog B (C. elegans)	60630	2.06E-21	20	28165595	28166390	795
Lcn11	lipocalin 11	100169711	3.73E-15	3	3888286	3888886	600
Lamc3	laminin gamma 3	311862	1.11E-21	3	10985872	10986472	600
Olr425	olfactory receptor 425	296687	1.05E-09	3	16610744	16611459	715
Serf2	small EDRK-rich factor 2	502663	4.86E-14	3	108254804	108255580	776
Vom1r102	vomeronasal 1 receptor 102	286957	9.69E-08	4	124241988	124242588	600
Ppap2b	phosphatidic acid phosphatase type 2B	192270	4.56E-31	5	126121326	126122026	700
Ctrc	chymotrypsin C (caldecrin)	362653	2.41E-27	5	160755960	160756730	770
Clip4	CAP-GLY domain containing linker protein family, member 4	298801	3.02E-17	6	23831232	23832132	900
Olr1016	olfactory receptor 1016	288858	6.29E-25	7	6876471	6877071	600
Rabl2b	RAB, member of RAS oncogene family-like 2B	362987	1.17E-28	7	127910262	127910992	730
Nfkbie	nuclear factor of kappa light polypeptide gene enhancer in B-cells inhibitor, epsilon	316241	5.02E-12	9	11053187	11053787	600
Aox3	aldehyde oxidase 3	493909	1.65E-14	9	56779670	56780578	908
Rpl39	ribosomal protein L39	25347	9.66E-17	X	7824369	7825465	1096
Nxf7	nuclear RNA export factor 7	501621	8.46E-20	X	122897736	122898626	890

Previously the sperm DMR identified in vinclozolin lineage F3 generation animals was reported [16]. An overlap of these sperm DMR with the current granulosa cell DMR demonstrated no overlapped sites. The lack of DMR overlap demonstrates different transgenerational epigenomes between the sperm and granulosa cell. It is anticipated that the cascade of epigenetic and transcriptome steps to achieve a differentiated somatic cell will lead to very distinct cell specific epigenomes with minimal overlap with the germ line [14]. Therefore, observations demonstrate that the F3 generation vinclozolin lineage granulosa cells have transgenerational changes in the epigenome that correlate with transgenerational changes in the transcriptome that in turn are proposed to have a role in the induction of the transgenerational ovarian disease. All the other cell types in the ovary (e.g. oocyte, theca cells, ovarian stromal cells) are also expected to have transgenerational epigenome and transcriptome changes that will also contribute to ovarian disease. The granulosa cell observations provide the proof of concept that the transgenerational disease phenotype develops from the transgenerational effects of the altered epigenome on somatic cell transcriptomes.

## Discussion

The most common human diseases of the ovary are primary ovarian insufficiency and polycystic ovarian disease. These conditions can cause infertility and increase the risk for other related health issues. Primary ovarian insufficiency affects about 1% of women, while polycystic ovarian disease affects as many as 18% of women [25], [26], [27], [37], [38], [39], [40], [41], [42], [44]. In the current study, F0 generation gestating female rats were exposed to various environmental compounds during fetal gonadal sex determination followed by F1 and F3 generation progeny being examined for ovarian histology. Ovarian abnormalities resembling the follicle pool depletion that precedes primary ovarian insufficiency and the cyst formation of polycystic ovarian disease were observed transgenerationally at an increased rate in the F3 generation exposure lineage animals. Molecular studies were performed comparing F3 generation control to vinclozolin lineage animals that indicated that there were transgenerational alterations in the epigenome and transcriptome of granulosa cells from ovarian follicles. These results raise the possibility that the disease etiology may in part be a result of exposure to environmental toxicants that promote epigenetic transgenerational inheritance of ovarian disease.

The analysis of ovarian follicle counts showed that there were significantly fewer oocytes in the ovaries of all of the exposure lineage females. Mean decreases in primordial follicle counts of 35% to 60% were seen in both the F1 and the F3 generation animals (Fig. 1). Since F1 generation animals were directly exposed to the environmental compounds *in utero* when the F0 gestating females were exposed, the F1 generation decrease in oocyte number compared to controls can be due to direct exposure of the follicles to the compounds. This possibility was tested using an organ culture system in which ovaries isolated from neonatal rats were treated with varying doses of vinclozolin or were left untreated as controls. A dose of 500 µM vinclozolin resulted in significantly fewer oocytes, while 200 µM and lower concentrations were not significantly different from controls (Fig. 2). F0 generation gestating female rats were treated with 100 mg/kg vinclozolin, which converts to approximately a 350 µM dose (assuming a whole-body volume of distribution). So it is conceivable that germ cells/oocytes could be lost in the F1 females when their F0 generation mothers are treated with vinclozolin. The epigenetic transgenerational inheritance of adult onset disease induced by the toxicants used in previous studies [19], [78] demonstrates that the compounds or their metabolites pass the placenta to reach the fetus. Direct exposure to several of the toxicants used in this study has previously been shown to affect ovarian function and development. Neonatal exposure to BPA has been shown to decrease the pool of primordial follicles and increase the incidence of ovarian cyst formation in rat ovaries, similarly to results of the current study [79], [80]. Exposure to the plastic phthalate DEHP was shown to inhibit steroidogenesis in rat granulosa cells [81], and to impair oocyte growth and ovulation in zebrafish [82]. Female rats treated with dioxin both during fetal development and after birth were reported to have a premature cessation of reproductive cycles as they aged [65]. However, these direct exposure effects cannot explain the significant decrease in oocyte number seen in F3 generation exposure lineage females. F3 generation females were not directly exposed to the environmental compounds, which suggests a potential epigenetic transgenerational inheritance molecular mechanism.

Primary ovarian insufficiency in humans is characterized by an early loss of ovarian follicles and onset of menopause. This can occur when the pool of oocytes in the ovary is depleted to less than 1% of the amount present at puberty, as occurs with menopause [83], [84]. The major physiological parameter of POI is the loss of the primordial follicle pool. In the current study, F1 and F3 treated-lineage groups of animals showed a 35–60% decrease in primordial follicle numbers compared to age-matched one-year-old controls. Approximately 45% of all the exposure lineage F3 generation females developed follicle pool depletion. Normal female rats usually stop cycling and enter reproductive senescence at 15–18 months of age. Other studies have shown that experimental depletion of oocyte numbers in rodents leads to an early loss of reproductive cycles [85], [86], [87]. Therefore, it is expected that animals from toxicant exposure lineages with follicle pool depletion would have a higher incidence of premature reproductive senescence (e.g. POI), but this remains to be investigated.

Polycystic ovarian disease is characterized by multiple persistent ovarian cysts [43]. In the current study increased numbers of ovarian cysts were seen in all the treated-lineage groups compared to the control lineage groups (Fig. 3). Interestingly, this effect was much more pronounced in the transgenerational F3 animals than in the directly exposed F1 generation. This suggests that the PCO disease identified may be due primarily to epigenetic transgenerational mechanisms and not to direct exposure. In addition, PCO disease was primarily observed in the 1 year old animals and not in young adults of 120 days of age [19], which is similar to what is observed in humans. An increase in circulating androstenedione was observed in the F3 generation vinclozolin lineage females that had PCO, similar to the clinical phenotype in women with PCO. Interestingly, research has shown that androstenedione levels are also increased in animals with follicle pool depletion [88]. The large cysts found in environmental exposure lineage females (vinclozolin, pesticide, jet-fuel and low-dose plastics groups) often had a negligible layer of epithelial/granulosa cells lining the cavity and only a stromal/thecal layer surrounding the cyst. These resemble the follicular cysts in PCOS patients [89], [90], [91].

Luteal cysts were found only in the jet-fuel exposure lineage animals. These are cysts thought to form in the center of *corpora lutea* and are characterized by their surrounding band of luteal cells. This finding suggests that treatment with the different environmental toxicants can result in different transgenerational phenotypes. In contrast, all the different toxicant treatments resulted in the same increase in small ovarian cysts and in the same decrease in the primordial follicle pool. This is the case even though the different environmental exposures used are chemically dissimilar and would be expected to act through different signaling mechanisms. Observations suggest that some physiological processes in the ovary may be more prone to dys-regulation, independent of the environmental insult. For example, the complex signaling network that maintains primordial follicles and their oocytes in an arrested state [70], [74], [92], [93] may be sensitive to dys-regulation that then leads to accelerated loss of follicles and oocytes from the ovary. Further research into this environmentally induced epigenetic transgenerational inheritance model is needed to determine the specific etiologies of adult onset ovarian diseases.

### Molecular Etiology of Transgenerational Adult Onset Ovarian Disease

All the environmental exposures used in the current study induced transgenerational ovarian abnormalities. From among these exposures vinclozolin was used as a toxicant to study the molecular changes occurring transgenerationally in granulosa cells. Although all ovarian cell types (e.g. oocytes) are anticipated to develop a transgenerational alteration in the transcriptome and epigenome, granulosa cells were selected to provide the proof of concept for this phenomena. Vinclozolin is an agricultural fungicide with anti-androgenic endocrine disrupting activity [94]. Several studies have shown transgenerational effects following exposure of gestating rats during the period of fetal gonadal sex determination to vinclozolin [13]. These effects in the F3 generation animals include increased incidence of adult onset diseases such as cancer, kidney disease, immune abnormalities, prostate disease, spermatogenic defects and infertility [20], [75], [78], [95], [96]. F3 generation female rats after ancestral vinclozolin exposure have been shown to have uterine bleeding abnormalities late in pregnancy [75]. The molecular mechanism involved in epigenetic transgenerational inheritance requires an epigenetic alteration in the germline (egg or sperm) to transmit the phenotype [13]. An environmental exposure during fetal gonad sex determination appears to be required due to the epigenetic programming of primordial germ cells during this developmental period. Previous research with vinclozolin lineage rats has shown that permanent alterations in the male germ line epigenome are transmitted to subsequent generations and do not get erased after fertilization during early embryonic development, in a manner similar to imprinted genes [16]. A recent study demonstrates all the exposures used in the current study promote exposure specific epigenetic transgenerational alterations in the sperm epigenome [19]. Since the altered base-line epigenome of the sperm promotes an altered epigenome in cells and tissues that develop from that sperm, all tissues, including the ovary, are anticipated to have altered genome activity and develop a susceptibility to develop disease [76].

In the current study granulosa cells from large antral follicles of F3 generation females were evaluated for differences in either the gene expression profile or the epigenetic pattern of vinclozolin-lineage granulosa compared to controls. The gene expression of granulosa cells from F3 generation vinclozolin and control lineage animals was evaluated by microarray analysis. More than 500 genes were found to be differentially expressed compared to controls (Table S1). This is a transgenerational effect of the environmental compound exposure. The altered gene expression profile of vinclozolin lineage granulosa cells could contribute to the adult-onset development of abnormalities such as primary ovarian insufficiency or polycystic ovarian disease. Bioinformatic analysis of the differentially expressed gene list indicated that certain well-characterized cellular pathways and processes could be affected by changes in these genes (Table 1, Figure S2a, S2b). Interestingly, many genes involved in lipid metabolism and steroid precursor synthesis had altered expression, and this has been shown to potentially be involved in the pathology of polycystic ovarian disease [43]. Analysis of genes present within the 523 differentially expressed gene set that have previously been correlated to ovarian disease revealed 30 genes, Figure 7. In addition, 5 genes have been shown to be directly correlated to polycystic ovarian disease. Therefore, the current study involving an environmental toxicant induced epigenetic transgenerational inheritance of adult onset ovarian disease also identified a number of genes previously shown to be associated with ovarian disease. A previous study compared the transcriptomes of cumulus granulosa cells from human PCOS patients and normal control women after culturing the cumulus cells. Similarly to the current study, genes in the MAPK signaling pathway and in extracellular matrix/cell adhesion were found to be affected. However, few specific differentially expressed genes were found in common with the current study [97].

A gene network analysis of the transgenerationally altered granulosa cell transcriptome generated a highly connected set of potential regulatory genes (Fig. 6) associated with the ovarian abnormalities identified. This regulatory gene network provides potential new therapeutic targets and diagnostic markers to consider in ovarian disease etiology. Critical gene targets for future studies to be considered include *Esr1, Esr2, Srebf2, Mmp2, Cxcl12, Lpl, Stat5b* and *Hmgcr*.

The F3 generation granulosa cell epigenome analysis of differential DNA methylation demonstrated 43 different DMR in promoters. The MeDIP-Chip analysis used a comparative hybridization of F3 generation granulosa cell DNA for vinclozolin versus control lineage animals to increase sensitivity. A promoter tiling array Chip was used, so the majority of the genome was not examined. Therefore, the 43 DMR identified in promoters are a sub-set of the total epigenetic modifications possible. The anticipation is a larger set of epigenetic modifications are present genome wide. The 43 DMR identified in the F3 generation granulosa cell epigenome demonstrates an environmental induced transgenerational alteration that is correlated to the onset of ovarian abnormalities. The DMR were present on most autosomes. The CpG content of these DMR was 1–10 CpG per 100 bp. Previously, low density CpG regions have been shown to be involved in epigenetic transgenerational alterations in sperm [19], [98]. This genomic feature is speculated to be important in the epigenetic programming mechanism [19]. Interestingly, only one DMR (*Plekhm1*) was present in the promoter of one of the transgenerational 523 differentially expressed granulosa cell genes. This is likely due to a random overlap. Therefore, a relatively low number of epigenetic DMR sites could not explain the relatively large number of differentially expressed genes observed. Direct epigenetic regulation of individual promoters appears not to be involved. The hypothesis developing is that the epigenetic regulatory sites associated with the DMR may influence distal gene expression through non-coding RNA and are termed epigenetic control regions (ECR). This is similar on a molecular level to the imprinting control regions (ICR) previously identified (e.g. IGF2 and H19) [77]. The transgenerational differentially expressed gene set of 523 genes was examined in regards to chromosomal location and 26 gene clusters of 2–5 Mbase were identified with a statistically significant (p<0.05) over-represented set of genes, Figure 8 and Table S2. Several of these gene clusters correlated to the location of a DMR (approximately 15%). In addition, the small number (i.e. 20) of characterized rat long non-coding RNA (lncRNA) had 3 sites that overlapped with the ECR, but further characterization of the rat lncRNA's is required before functional associations between ECR and lncRNA can be elucidated. Future studies will be needed to determine the functional significance of these potential ECR sites, but the current study suggests the potential presence of such sites. The potential presence of DMR regulating such an ECR is speculated to clarify how a limited number of alterations in the epigenome may influence a large number of differentially expressed genes.

The molecular factors involved in epigenetic regulation of genome activity (i.e. DNA methylation, histone modifications, chromatin structure and non-coding RNA) can all regulate proximal promoter activity and gene regulation. Epigenetic factors such as DNA methylation, chromatin structure and non-coding RNA can also regulate distal gene expression, independent of classic genetic mechanisms. In the current study environmentally induced transgenerational effects on the germ line promoted epigenome and transcriptome effects in the granulosa cell that correlate with adult onset ovarian abnormalities. The etiology of ovarian diseases such as PCO and POI appear to in part involve: 1) environmental toxicant induced epigenetic alterations in the germ-line (sperm) during fetal gonadal development; 2) permanent alterations in the epigenome that are transmitted to subsequent generations through the sperm; 3) induction of alterations in the epigenome and transcriptome of all organs, such as the ovary, and cells such as granulosa cells; and 4) an increased susceptibility to develop adult onset ovarian disease such as polycystic ovarian disease or primary ovarian insufficiency. Although the current study establishes the proof of concept such a mechanism exists, the degree the environment and epigenetic transgenerational inheritance is involved in human ovarian disease now needs to be investigated. Future studies are needed to clarify the F1, F2 and F3 generation sperm epigenome alterations in relation to each other, functional links of the DMR with the lncRNA and ECR, and to characterize transgenerational developmental changes in the transcriptome and epigenome of all ovarian cell types (e.g. oocyte, granulosa and theca). Elucidation of these molecular processes and mechanism will provide insights into the molecular etiology of ovarian disease.

### Summary

An outbred rodent rat model was used to investigate the potential role of environmental epigenetics and epigenetic transgenerational inheritance in the etiology of ovarian disease. One of the ovarian abnormalities observed involved a decrease in the ovarian follicle pool size which correlates with the biology of primary ovarian insufficiency. The development of small and large ovarian cysts and the morphology of the cysts correlates with the biology of polycystic ovarian disease. However, these ovarian diseases as defined in humans are broader in concept to include correlated endocrine abnormalities and associated disease such as insulin resistance and diabetes. Therefore, the current ovarian abnormalities and disease in rats cannot be directly correlated to the human polycystic ovarian syndrome (PCOS) nor human primary ovarian insufficiency (POI) and loss of fertility. Although the rat ovarian abnormalities are consistent with these disease states, further research will be needed to clarify the role of environmental epigenetics and epigenetic transgenerational inheritance of ovarian disease in humans. Elucidation of such a disease etiology could help provide insight into clarifying the rapid increase in incidence of ovarian disease and apparent environmental impacts.

The environmental toxicants vinclozolin, dioxin and bisphenol-A have been shown in previous research to have transgenerational effects [21], [22], [75], [78]. In recent research from our laboratory, all of the environmental toxicants used in the current study were shown to cause transgenerational disease in rats [19]. In the current study DEHP and DBP were used in combination with bisphenol-A as a single treatment, so it is uncertain if alone the compounds can promote transgenerational disease in ovaries. Similarly, permethrin was used in combination with DEET, so evidence suggests the mixture can promote epigenetic transgenerational disease. The hydrocarbon mixture jet fuel (JP8) also promoted a transgenerational increase in the incidence of ovarian disease in these studies. Results suggest that all these compounds should now be considered as potentially able to promote transgenerational ovarian disease.

The current study used pharmacologic doses of all the compounds and mixtures based on approximately 1% of the oral LD50 dose for most compounds, Table S3. The objective was to determine if these exposures have the capacity to promote epigenetic transgenerational inheritance of a disease phenotype, and not to do risk assessment of the exposures. Now that the current study has established the transgenerational actions of these compounds, risk assessment toxicology involving dose curves of relevant environmental doses are needed. In addition to considering the mode of administration and dose, the critical window of exposure to promote the epigenetic transgenerational phenotype (gonadal sex determination) needs to be considered, which for the human is 6–18 weeks of gestation. The gestating women in the first half of pregnancy would be the population most sensitive to environmentally induced epigenetic transgenerational inheritance of disease phenotypes.

In summary, gestating F0 generation rats were treated with environmental toxicants transiently during fetal gonadal sex determination. Adult-onset ovarian diseases resembling primary ovarian insufficiency and polycystic ovarian disease were seen at an increased rate in both the directly exposed offspring (F1), and transgenerationally (F3). There was a significant transgenerational alteration in both the transcriptome and the epigenome of vinclozolin-lineage granulosa cells. Therefore, ancestral toxicant exposure can contribute to the development of these disease states. These results suggest a new paradigm be considered for the etiology of ovarian disease. In addition to genetic abnormalities being causative, epigenetic abnormalities can also cause changes in gene expression during development that lead to these adult-onset diseases. These epigenetic abnormalities can be induced by exposure to a variety of environmental toxicants. If the exposure occurs during a susceptible period of an animal's development, then these epigenetic abnormalities can be fixed into the germ line (*i.e.* eggs or sperm) and be passed transgenerationally. Ovarian disease such as PCO has impacts on other diseases such as diabetes and adverse pregnancy outcomes [99]. Therefore, further elucidation of the etiology of ovarian disease and potential role of environmental epigenetics and epigenetic transgenerational inheritance will provide insights into the prevention and therapeutic strategies for female health.

## Methods

### Animals and treatments

All experimental protocols involving rats were pre-approved by the Washington State University Animal Care and Use Committee (IACUC approval # 02568-026). Hsd:Sprague Dawley®™SD®™ female and male rats of an outbred strain (Harlan) were maintained in ventilated (up to 50 air exchanges/hour) isolator cages (cages with dimensions of 10 ¾″W×19 ¼″D×10 ¾″H, 143 square inch floor space, fitted in Micro-vent 36-cage rat racks; Allentown Inc., Allentown, NJ) containing Aspen Sani chips (pinewood shavings from Harlan) as bedding, on a 14 h light: 10 h dark regimen, at a temperature of 70 F and humidity of 25% to 35%. Rats were fed ad libitum with standard rat diet (8640 Teklad 22/5 Rodent Diet; Harlan) and ad libitum tap water for drinking.

At proestrus as determined by daily vaginal smears, the female rats (90 days of age) were pair-mated with male rats (120 days). On the next day, the pairs were separated and vaginal smears were examined microscopically. If sperm were detected (day 0) the rats were tentatively considered pregnant. Vaginal smears were continued for monitoring diestrus status in these rats until day 7. Pregnant rats were then given daily intraperitoneal injections of any one of the following single chemicals or mixtures with an equal volume of sesame oil (Sigma) on days E-8 through E-14 of gestation [75], as seen in Table S3. Treatment groups were Control (DMSO vehicle), Vinclozolin, Pesticide/repellent (includes: Permethrin (insecticide) and DEET (insect repellent)), Plastics (Bisphenol-A, DBP and DEHP), Low-dose plastics, Dioxin (TCDD), and Jet Fuel (JP8 hydrocarbon). The pregnant female rats treated with various mixtures were designated as the F0 generation. A drop in litter size was noted in the F1 generation of the Plastics group, so another treatment group was included with only half the dose of Bisphenol-A, DBP and DEHP and this group was designated the ‘Low Dose Plastics’ group. Doses, percent of oral LD50, and sources of the compounds are given in Table S3.

### Breeding for F1, F2, and F3 generations

The offspring of the F0 generation were the F1 generation. The F1 generation offspring were bred to other F1 animals of the same treatment group to generate an F2 generation and then F2 generation animals bred similarly to generate the F3 generation animals. No sibling or cousin breedings were performed so as to avoid inbreeding. Note that only the original F0 generation pregnant females were injected with the treatment compounds.

### Evaluation of adult ovaries

Ovaries taken from rats at the time of sacrifice (one year of age) were fixed in Bouin's solution, paraffin embedded and sectioned at 5 µm thickness. Every 30^th^ section was collected and hematoxylin/eosin stained. The three stained sections (150 µm apart) through the central portion of the ovary with the largest cross-section were evaluated microscopically for number of primordial follicles, developing pre-antral follicles, small antral follicles, large antral follicles, small cystic structures and large cysts. The mean number of each evaluated structure per section was calculated across the three sections. Follicles had to be non-atretic and have the oocyte nucleus visible in the section in order to be counted. Atretic follicles have granulosa cells or oocytes with pyknotic nuclei, an uneven or reduced layer of granulosa cells, and/or an uneven and less distinct basement membrane. Primordial follicles are in an arrested state and have an oocyte surrounded by a single layer of either squamous or both squamous and cuboidal granulosa cells [100], [101]. Normally a few primordial follicles at a time will undergo primordial to primary follicle transition and become developing follicles. Developing pre-antral follicles had one or more complete layers of cuboidal granulosa cells. Small antral follicles had a fluid-filled antrum and a maximum diameter of 510 µm measured across the outermost granulosa cell layer. Large antral follicles had a diameter greater than 510 µm. Large antral follicles may eventually ovulate. Cysts were defined as fluid-filled structures of a specified size that were not filled with red blood cells and which were not follicular antra. A single layer of cells may line cysts. Small cysts were 50–250 µm in diameter measured from the inner cellular boundary across the longest axis. Large cysts were greater than 250 µm in diameter.

### Neonatal rat ovary culture

Four-day old female Sprague-Dawley rats (Harlan Laboratories, Inc., USA) were euthanized according to Washington State University IACUC approved protocols and their ovaries removed and cultured whole as described previously [102]. Four-day old rat ovaries contain follicles that are almost exclusively of the primordial stage. Whole ovaries were cultured on floating filters (0.4 µm Millicell-CM, Millipore, Bedford, MD, USA) in 0.5 ml Dulbecco's modified Eagle's medium (DMEM)-Ham's F-12 medium (1∶1, vol/vol) containing 0.1% BSA (Sigma), 0.1% Albumax (Gibco BRL, Gaithersburg, MD, USA), 27.5 µg/ml transferrin, and 0.05 mg/ml L-ascorbic acid (Sigma) in a four-well culture plate (Nunc plate, Applied Scientific, South San Francisco, CA, USA) for ten days. Previous studies have shown that four-day-old ovaries cultured for ten days have good cell viability [103]. The medium was supplemented with penicillin and streptomycin to prevent bacterial contamination. Ovaries were randomly assigned to treatment groups, with 1–3 ovaries per floating filter per well. Culture medium was changed and wells were treated every two days with vinclozolin (50 µM, 100 µM, 200 µM, or 500 µM), or were treated with 0.1% DMSO as a vehicle control. After culture, ovaries were fixed in Bouin's fixative (Sigma) for two hours and then equilibrated in 70% ethanol. Ovaries were then embedded in paraffin, sectioned at 3 µm and stained with hematoxylin/eosin for use in morphological analysis.

For each ovary the number of oocytes per section was counted and the counts were averaged across the two consecutive histological sections that had the largest ovarian cross section. The oocyte nucleus had to be visible for an oocyte to be counted. Normally, between 50 and 150 follicles were present in each cross-section.

Blood samples were collected, allowed to clot, centrifuged and serum samples stored for hormone assays. The androstenedione levels in serum were determined with a radio-immunoassay (RIA) performed by the Center for Reproductive Biology Assay Core at Washington State University.

### Super-ovulation and collection of granulosa cells

F3 generation rats from both vinclozolin-treated and control lineages were treated with Pregnant Mare Serum Gonadotropin (Sigma cat, St. Louis, MO)(30 IU PMSG injected IP) at five to six months of age. Two days later animals were sacrificed and ovaries removed. The ovarian bursa and its adherent fat was removed from each ovary and the ovaries processed for granulosa cell collection [104]. The ovaries were suspended in the base medium used for all procedures was Ham's F-12 (Thermo Scientific). Following sequential 30 minute incubations at 37 °C in 6 mM EGTA in F-12 (to decrease Ca^2+^ - mediated cell adhesion) and then 0.5 M sucrose in F-12 (to increase osmotic pressure within follicles), ovaries were returned to F-12. Granulosa cells were released into the medium from antral follicles using 30-gauge needles and gentle pressure. Oocytes were removed by aspiration under a dissecting microscope. Granulosa cells from each rat were collected into 1.5 mL tubes, allowed to settle for 10 minutes and supernatant removed. 1.0 mL of Trizol™ (Invitrogen) was added to each sample, and then samples were stored at −70° until the time of RNA and DNA isolation.

### Microarray transcriptome analysis

Messenger RNA was isolated from Trizol™ for each animal as per manufacturers protocol. Messenger RNA from four animals of the same treatment group were pooled to create three different pooled samples from each of the two treatment groups. The mRNA processing and hybridization were performed at the Genomics Core Laboratory, Center for Reproductive Biology, Washington State University, Pullman, WA using standard Affymetrix reagents and protocol. Briefly, mRNA was transcribed into cDNA with random primers, then cRNA was transcribed from the cDNA, and from that, single-stranded sense DNA was synthesized which was fragmented and labeled with biotin. Biotin-labeled fragmented ssDNA was then hybridized to the Rat Gene 1.0 ST microarrays containing more than 27,000 transcripts (Affymetrix, Santa Clara, CA, USA). Hybridized chips were scanned on an Affymetrix Scanner 3000. CEL files containing raw data were then pre-processed and analyzed with Partek Genomic Suite 6.5 beta software (Partek Incorporated, St. Louis, MO) using an RMA and GC-content adjusted algorithm (Figure S1). The signals from an average of 28 different probes for each transcript were compared to give a single value. Lists of differentially expressed genes for each treatment were generated using the following cut off criteria: signal ratio Treatment/Control >1.20 change, mean difference for un-logged signals between control and treatment >10, t-test p-values<0.05 using an analysis correcting for organ culture date batch effects.

CEL files from this study have been deposited with the NCBI gene expression and hybridization array data repository (GEO, http://www.ncbi.nlm.nih.gov/geo, #GSE 33423) and can be also accessed through www.skinner.wsu.edu. For gene annotation, Affymetrix annotation file RaGene1_0stv1.na31.rn4.transcript.csv was used unless otherwise specified.

To look for known functional relationships among the F3 generation differentially expressed genes identified above, KEGG pathways were interrogated using the http://www.genome.jp/kegg/ website (Kyoto Encyclopedia for Genes and Genome, Kyoto University, Japan), and also using Pathway Express, a web-based tool freely available as part of the Onto-Tools website (http://vortex.cs.wayne.edu) [105].

To further look for known functional relationships among the F3 differentially expressed genes, an unbiased, automated survey of published literature was performed to determine which genes are functionally linked with respect to binding, up-regulation, down-regulation, *etc.* Global literature analysis of differentially expressed genes was performed using Pathway Studio software (Ariadne, Genomics Inc. Rockville MD), which performs an interaction analysis and builds sub-networks of genes and the cellular processes that connect them to each other.

Previous studies have demonstrated that microarray data are validated with quantitative PCR data [106], [107]. Due to the presence of an average of 28 different oligonucleotide probes for each specific gene being used on the microarray versus only a single primer set for a gene in a quantitative PCR, the microarray is more effective at eliminating false positive or negative data and provides a more robust quantification of changes in gene expression.

### Methylated DNA immunoprecipitation (MeDIP)

DNA was collected from the same granulosa cell Trizol™ preparations that were used for RNA isolation. The DNA Trizol™ fractions from four animals of the same treatment group were pooled to create three different pooled DNA samples from each of the two treatment groups. These DNA samples were then used for methylated DNA immunoprecipitation (MeDIP). MeDIP was performed as follows: 6 µg of genomic DNA was subjected to series of three 20 pulse sonications at 20% amplitude and the appropriate fragment size (200–1000 bp) was verified through 2% agarose gels; the sonicated genomic DNA was resuspended in 350 µl TE and denatured for 10 min at 95°C and then immediately placed on ice for 5 min; 100 µl of 5× IP buffer (50 mM Na-phosphate pH7, 700 mM NaCl, 0.25% Triton X-100) was added to the sonicated and denatured DNA. An overnight incubation of the DNA was performed with 5 µg of antibody anti-5-methylCytidine monoclonal from Diagenode S.A (Denville, NJ) at 4°C on a rotating platform. Protein A/G beads from Santa Cruz (Santa Cruz, CA) were prewashed on PBS-BSA 0.1% and resuspended in 40 µl 1× IP buffer. Beads were then added to the DNA-antibody complex and incubated 2 h at 4°C on a rotating platform. Beads bound to DNA-antibody complex were washed 3 times with 1 ml 1× IP buffer; washes included incubation for 5 min at 4°C on a rotating platform and then centrifugation at 6000 rpm for 2 min. Beads-DNA-antibody complex were then resuspended in 250 µl digestion buffer (50 mM Tris HCl pH 8, 10 mM EDTA, 0.5% SDS) and 3.5 µl of proteinase K (20 mg/ml) was added to each sample and then incubated overnight at 55°C on a rotating platform. DNA purification was performed first with phenol and then with chloroform:isoamyl alcohol. Two washes were then performed with 70% ethanol, 1 M NaCl and glycogen. MeDIP selected DNA was then resuspended in 30 µl TE buffer. Whole-genome amplification was then performed with the WGA2 kit (Sigma-Aldrich #WGA2) on each MeDIP sample to be used in the microarray comparative hybridization analysis.

### Tiling Array MeDIP-Chip Analysis

Roche Nimblegen's Rat DNA Methylation 3x720K CpG Island Plus RefSeq Promoter Array was used, which contains three identical sub-arrays, with 713,670 probes per sub-array, scanning a total of 15,287 promoters (3,880 bp upstream and 970 bp downstream from transcription start site). Probe sizes range from 50–75 mer in length with a median probe spacing of 100 bp. Three different comparative (amplified MeDIP vs. amplified MeDIP) hybridizations experiments (3 sub-arrays) were performed, each encompassing DNA samples from 24 animals (3 treatment and 3 control groups). MeDIP DNA samples from experimental groups were labeled with Cy3 and MeDIP DNA samples from the control groups were labeled with Cy5.

### Bioinformatic and Statistical Analyses of Chip Data

For each comparative hybridization experiment, raw data from both the Cy3 and Cy5 channels were imported into R (R Development Core Team (2010), R: A language for statistical computing, R Foundation for Statistical Computing, Vienna, Austria. ISBN 3-900051-07-0, URL http://www.R-project.org), checked for quality and converted to MA values (M = Cy5−Cy3; A = (Cy5+Cy3)/2). The following normalization procedure was conducted. Within each array, probes were separated into groups by GC content and each group was separately normalized, between Cy3 and Cy5 using the loess normalization procedure. This allowed for GC groups to receive a normalization curve specific to that group. After each array had its CG groups normalized within the array, the arrays were then normalized across arrays using the A quantile normalization procedure.

Following normalization each probe within each array was subjected to a smoothing procedure, whereby the probe's normalized M values were replaced with the median value of all probe normalized M values across all arrays within a 600 bp window. If the number of probes present in the window was less than 3, no value was assigned to that probe. Each probe's A values were likewise smoothened using the same procedure. Following normalization and smoothing each probe's M value represents the median intensity difference between vinclozolin generation and control generation of a 600 bp window. Significance was assigned to probe differences between treatment-generation samples and control generation samples by calculating the median value of the intensity differences as compared to a normal distribution scaled to the experimental mean and standard deviation of the normalized M. A Z-score and P-value were computed for each probe from that distribution. The statistical analysis was performed in pairs of comparative IP hybridizations between treatment lineage (T) and control lineage (C). T1-C1 and T2-C2 gave 333 sites; T1-C1 and T3-C3 gave 327 sites; T2-C2 and T3-C3 gave 340 sites. In order to assure the reproducibility of the candidates obtained, only the candidates showing significant changes in all three of the paired comparisons were chosen as having a significant change in DNA methylation between the experimental group and controls. This is a very stringent approach to select for changes, since it only considers those changes repeated in all paired analyses.

Clustered Regions of interest were then determined by combining consecutive probes within 600 bases of each other, and based on whether their mean M values were positive or negative, with significance p-values less than 10^−5^. The statistically significant differential DNA methylated regions were identified and P-value associated with each region presented. Each region of interest was then annotated for gene and CpG content. This list was further reduced to those regions with an average intensity value exceeding 9.5 (log scale) and a CpG density ≥1 CpG/100 bp.

### Statistical Analysis for ovarian morphological data

Treatment groups are compared using analysis of variance (ANOVA) followed by Dunnet's post-hoc tests where appropriate. Groups were considered statistically significant with P≤0.05. Statistics for ovary counts were calculated using Graph Pad Prism version 5.0 b for Macintosh, Graph Pad Software, San Diego, CA, USA.

## Supporting Information

Figure S1 **Sample histograms and box plots for granulosa cell microarray signal values after pre-processing with RMA, GC-content adjusted algorithm.** Plots for F3 generation control (red) and F3 generation vinclozolin (blue) microarrays.
(PDF)

Figure S2 **(A): Steroid Biosynthesis Pathway; and (B): PPAR Signaling Pathway showing granulosa cell differentially expressed genes between F3 generation vinclozolin and control lineage rats: red or red-countered boxes represent up-regulated genes, green down-regulated and white boxes – not affected genes.**
(PDF)

Table S1 **Rat granulosa cell genes differentially expressed between F3 generation vinclozolin and control lineage animals (523 genes).**
(PDF)

Table S2 **Differential expressed gene clusters.**
(PDF)

Table S3 **Doses and sources of chemicals used.**
(PDF)
