# Supplementary figures and images for: Environmentally Induced Epigenetic Transgenerational Inheritance of Ovarian Disease
Source: PLoS One. 2012 May 3;7(5):e36129. doi: 10.1371/journal.pone.0036129 (PMC3343040; doi:10.1371/journal.pone.0036129)

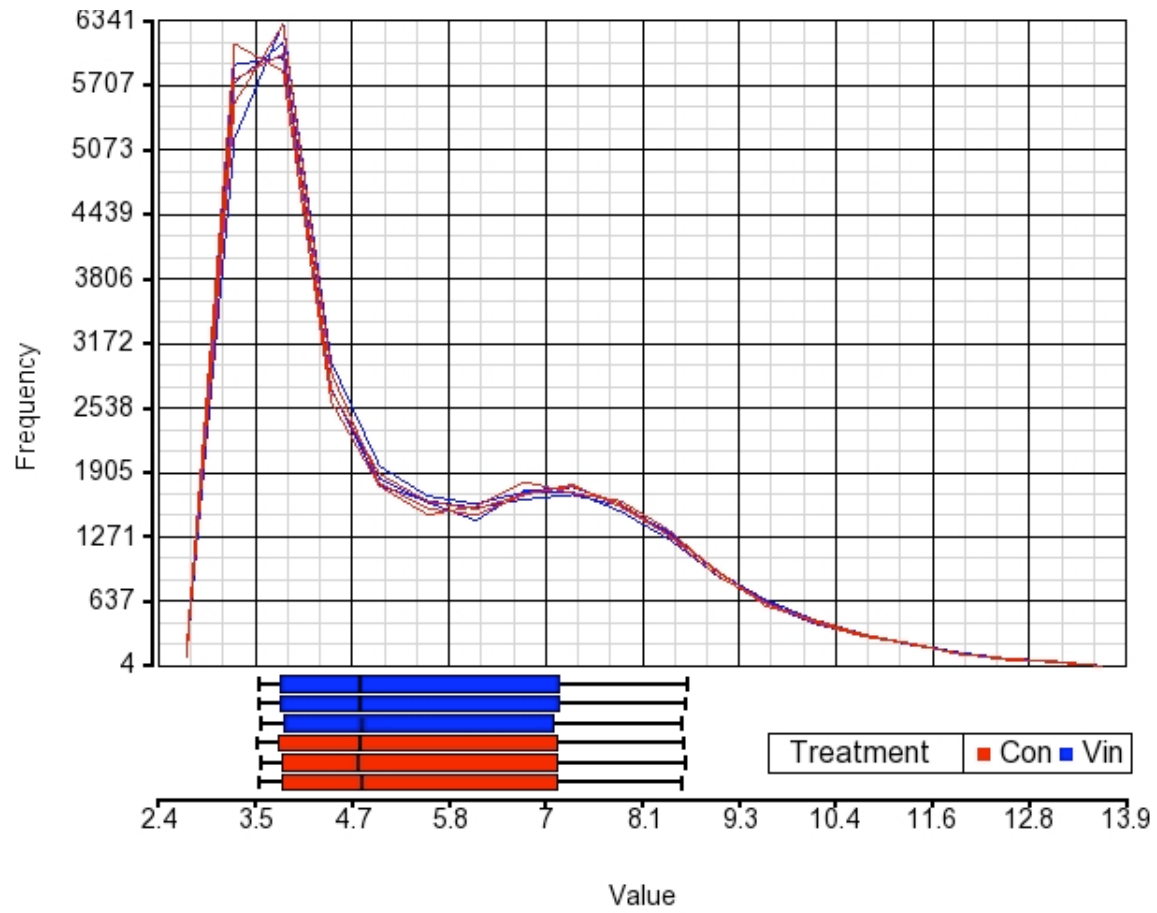

Supplemental Figure S1

Supplement: Figure S1 — Sample histograms and box plots for granulosa cell microarray signal values after pre-processing with RMA, GC-content adjusted algorithm. Plots for F3 generation control (red) and F3 generation vinclozolin (blue) microarrays. (PDF) [file pone.0036129.s001.pdf]
