# Supplementary material for: Environmentally Induced Epigenetic Transgenerational Inheritance of Ovarian Disease
Source: PLoS One. 2012 May 3;7(5):e36129. doi: 10.1371/journal.pone.0036129 (PMC3343040; doi:10.1371/journal.pone.0036129)
Supplement: Figure S2 — (A): Steroid Biosynthesis Pathway; and (B): PPAR Signaling Pathway showing granulosa cell differentially expressed genes between F3 generation vinclozolin and control lineage rats: red or red-countered boxes represent up-regulated genes, green down-regulated and white boxes – not affected genes. (PDF) [file pone.0036129.s002.pdf]

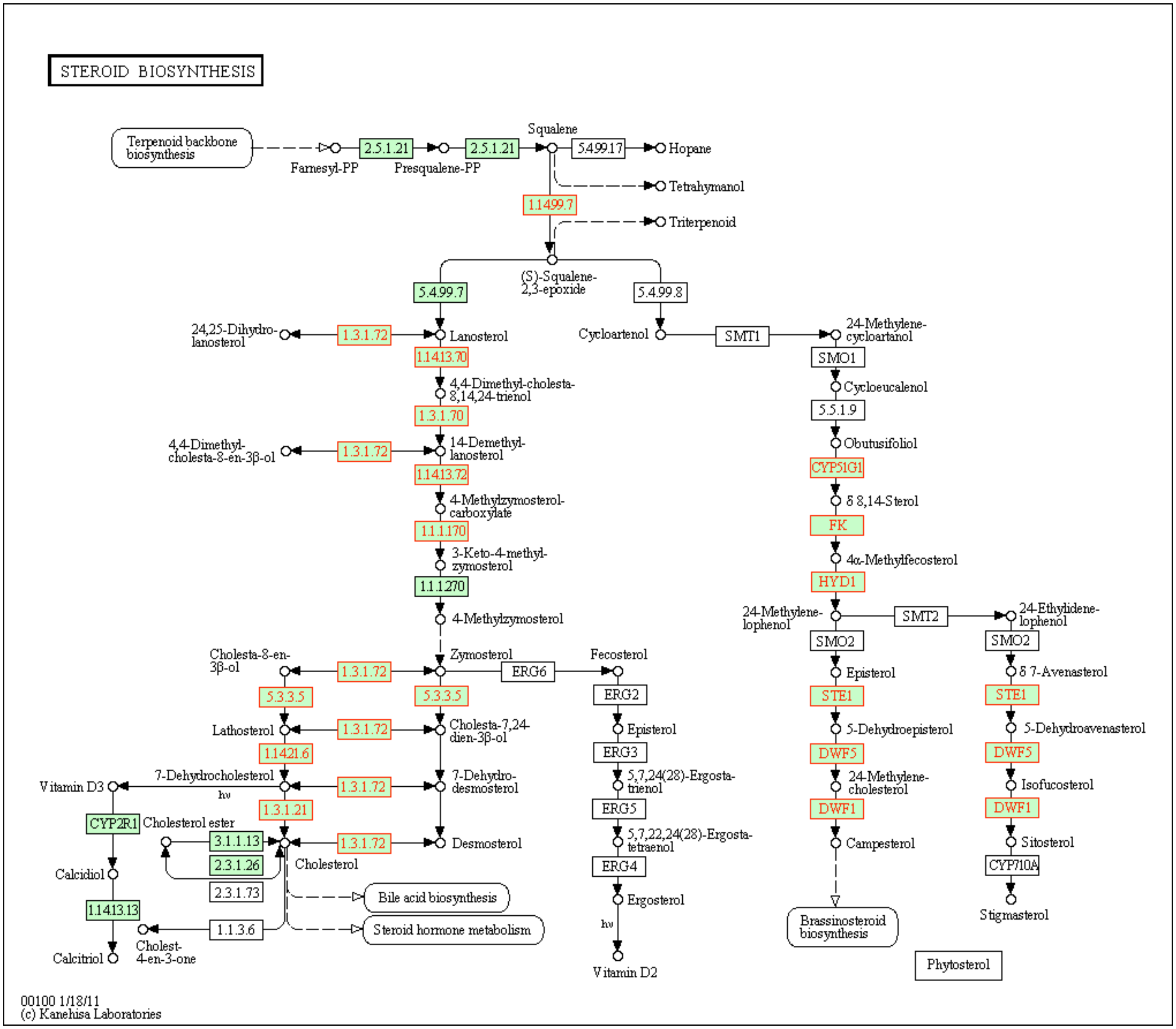

Supplemental Figure S2a

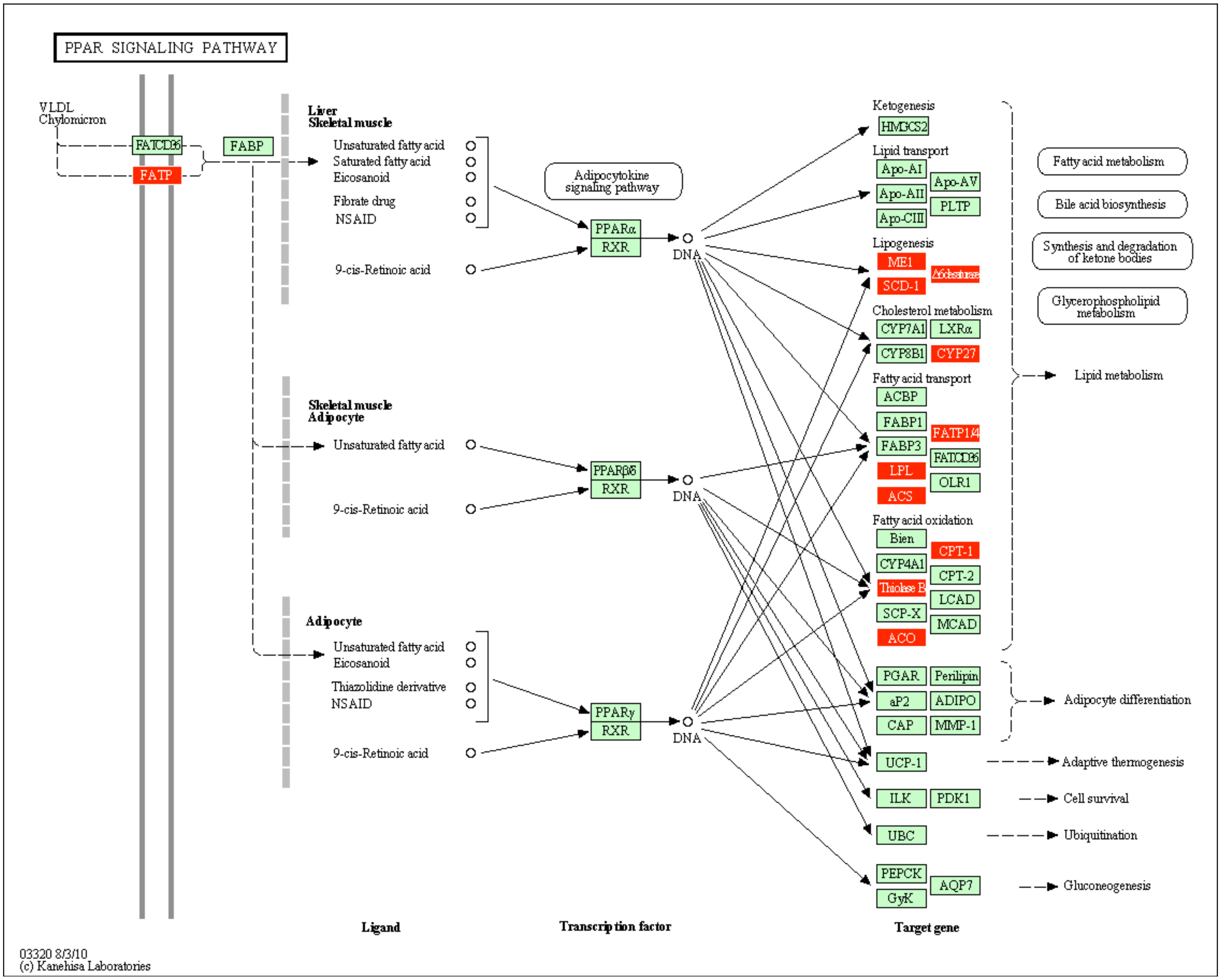

Supplemental Figure S2b
