# Supplementary material for: Environmentally Induced Epigenetic Transgenerational Inheritance of Ovarian Disease
Source: PLoS One. 2012 May 3;7(5):e36129. doi: 10.1371/journal.pone.0036129 (PMC3343040; doi:10.1371/journal.pone.0036129)
Supplement: Table S1 — Rat granulosa cell genes differentially expressed between F3 generation vinclozolin and control lineage animals (523 genes). (PDF) [file pone.0036129.s003.pdf]

Supplemental Table S1
Rat Granulosa Cell Genes differentially expressed between F3 generation vinclozolin and control lineage animals (523 genes)

Gene Symbol	GenBank, RefSeq Number	Vin/C Ratio	Vin-Con, mean_dif	Affymetrix Probeset ID	Gene Title
**Apoptosis**					
Apip	NM_001106492	1.22	13	10838145	APAF1 interacting protein
Ciapin1	NM_001007689	1.30	87	10805986	cytokine induced apoptosis inhibitor 1
Fbf1	NM_001105854	0.77	-16	10749142	Fas (TNFRSF6) binding factor 1
Nradd	NM_139259	1.35	64	10920461	neurotrophin receptor associated death domain
Tnrc6b	NM_138845	0.82	-46	10897821	trinucleotide repeat containing 6B
**Cell Cycle**					
Bop1	NM_001024250	1.28	40	10904930	block of proliferation 1
Cenpj	NM_001107265	0.73	-20.3	10784074	centromere protein J
Cep120	ENSRNOT0000002	0.76	-32	10804486	centrosomal protein 120kDa
Cspp1	XM_001060285	0.71	-69	10875087	centrosome and spindle pole associated protein 1
Fchsd2	NM_001107539	0.81	-27	10709135	FCH and double SH3 domains 2
Nek2	NM_053691	0.72	-38	10766765	NIMA (never in mitosis gene a)-related expressed kinase 2
Nubp2	NM_001011891	1.21	74	10741248	nucleotide binding protein 2
Pot1b	XM_001073777	0.79	-10	10931288	protection of telomeres 1B
**Cytoskeleton-ECM**					
Actn4	NM_031675	1.30	231	10720274	actinin alpha 4
Ankh	NM_053714	1.63	26	10813969	ankylosis, progressive homolog (mouse)
Arpc1b	NM_019289	1.44	104	10760196	actin related protein 2/3 complex, subunit 1B
Arpc3	NM_001105933	1.24	46	10762021	actin related protein 2/3 complex, subunit 3
Asam	NM_173154	1.73	36	10909338	adipocyte-specific adhesion molecule
Col4a3	NM_001135759	0.68	-15	10924719	collagen, type IV, alpha 3
Col6a1	ENSRNOT0000000	1.55	44	10829437	collagen, type VI, alpha 1
Cotl1	NM_001108452	1.51	22	10811452	coactosin-like 1 (Dictyostelium)
Csrp1	NM_017148	1.77	179	10764221	cysteine and glycine-rich protein 1
Dmkn	NM_001173357	1.88	40	10706065	dermokine
Epdr1	NM_001007625	1.37	30	10795384	ependymin related protein 1 (zebrafish)
F11r	NM_053796	2.45	103	10765600	F11 receptor
Kif1a	AM180765	1.84	39	10929937	kinesin family member 1A
Krtap4-5	ENSRNOT0000006	0.78	-21	10747180	keratin associated protein 4-5
Lgals3bp	NM_139096	1.71	149	10749495	lectin, galactoside-binding, soluble, 3 binding protein
LOC360570	NM_001172137	1.39	21	10736073	similar to myosin XVIIIa
Marcksl1	NM_030862	1.26	117	10872336	MARCKS-like 1
Myl12b	NM_017343	1.25	87	10930416	myosin, light chain 12B, regulatory
Nid67	NM_173126	1.54	58	10804562	putative small membrane protein NID67
RGD1563615	XM_001064593	0.61	-14	10793429	similar to Contactin associated protein-like 3 precursor (Cell recognition
Sdc1	NM_013026	1.64	66	10883530	syndecan 1
Tagln	NM_031549	2.02	134	10917034	transgelin
Tpm2	NM_001024345	1.86	31	10876324	tropomyosin 2, beta
Tubb6	NM_001025675	1.86	43	10802422	tubulin, beta 6
**Development**					
Atxn7l1	ENSRNOT0000001	0.78	-11	10884189	ataxin 7-like 1
Bean	XM_341638	1.69	80	10809044	brain expressed, associated with Nedd4
Cln8	NM_001007686	1.22	21	10792676	ceroid-lipofuscinosis, neuronal 8
Fhl3	NM_001107979	1.48	12	10871806	four and a half LIM domains 3
Flcn	NM_199390	1.23	23	10743171	folliculin

Jub	NM_053503	1.31	22	10783583	jub, ajuba homolog (Xenopus laevis)
LOC501092	ENSRNOT0000004	0.77	-12	10861620	hypothetical LOC501092
LOC680073	XR_086000	0.60	-55	10767160	similar to globin inducing factor, fetal
Npdc1	NM_001004231	1.37	35	10834225	neural proliferation, differentiation and control, 1
Rai2	NM_001109316	1.38	56	10937834	retinoic acid induced 2
Scrib	ENSRNOT0000004	0.81	-30	10904714	scribbled homolog (Drosophila)
Sesn1	NM_001106396	1.21	22	10830561	sestrin 1
Stim2	NM_001105750	0.80	-43	10776992	stromal interaction molecule 2
Suhw3	ENSRNOT0000004	0.75	-22	10939617	suppressor of hairy wing homolog 3 (Drosophila)
Syngr1	NM_019166	1.43	31	10897734	synaptogyrin 1
Ttyh2	ENSRNOT0000000	1.33	133	10739399	tweety homolog 2 (Drosophila)
Unc13b	NM_001042579	0.70	-19.9	10868437	unc-13 homolog B (C. elegans)
Vwa5a	NM_198755	1.65	124	10909210	von Willebrand factor A domain containing 5A
Wfs1	NM_031823	1.27	22	10773290	Wolfram syndrome 1 homolog (human)
Whsc1	XM_218310	0.75	-54	10704372	Wolf-Hirschhorn syndrome candidate 1 (human)
**DNA repair**					
RGD1561537	XM_001058999	0.74	-42	10796970	similar to putative repair and recombination helicase RAD26L
**Electron Transport**					
Cox5a	NM_145783	1.27	187	10910338	cytochrome c oxidase, subunit Va
Cyb5b	NM_030586	1.30	92	10807632	cytochrome b5 type B (outer mitochondrial membrane)
Cyb5r3	NM_138877	1.30	62	10905804	cytochrome b5 reductase 3
Cyp1b1	NM_012940	2.28	145	10887947	cytochrome P450, family 1, subfamily b, polypeptide 1
Cyp27a1	NM_178847	1.45	67	10924411	cytochrome P450, family 27, subfamily a, polypeptide 1
Cyp2t1	NM_134369	1.32	15	10705313	cytochrome P450, family 2, subfamily t, polypeptide 1
Cyp51	NM_012941	1.69	550	10853471	cytochrome P450, family 51
Fdxr	NM_024153	1.34	289	10748909	ferredoxin reductase
Prdx3	NM_022540	1.44	811	10731210	peroxiredoxin 3
Prdx5	NM_053610	1.23	77	10728240	peroxiredoxin 5
Prdx6	NM_053576	1.28	161	10769218	peroxiredoxin 6
**Epigenetics**					
Bhmt	NM_030850	2.14	66	10820494	betaine-homocysteine methyltransferase
Comtd1	NM_001107249	1.38	16	10778997	catechol-O-methyltransferase domain containing 1
H2afy2	NM_001135807	0.72	-34	10833093	H2A histone family, member Y2
Hmg1l1	NM_001109373	0.82	-413	10709775	high-mobility group (nonhistone chromosomal) protein 1-like 1
Hmgb1	NM_012963	0.77	-550	10902247	high mobility group box 1
Htatip2	NM_001106263	1.80	76	10707230	HIV-1 tat interactive protein 2, homolog (human)
Mettl7a	NM_001037355	1.34	23	10899263	methyltransferase like 7A
Smyd2	NM_206851	1.33	56	10770666	SET and MYND domain containing 2
Suv420h2	NM_001107475	0.82	-24	10718873	suppressor of variegation 4-20 homolog 2 (Drosophila)
Wdr77	NM_001008771	1.24	67	10818173	WD repeat domain 77
**Golgi Apparatus**					
Golga1	NM_001107842	0.83	-18	10844889	golgi autoantigen, golgin subfamily a, 1
Mpdu1	NM_001107011	1.22	77	10744119	mannose-P-dolichol utilization defect 1
Pigs	NM_001006602	1.20	51	10745081	phosphatidylinositol glycan anchor biosynthesis, class S
Pde6d	NM_001108806	1.22	66	10929600	phosphodiesterase 6D, cGMP-specific, rod, delta
**Growth Factors**					
Angptl2	NM_133569	1.33	23	10835654	angiopoietin-like 2
Creg1	NM_001105966	2.04	667	10765335	cellular repressor of E1A-stimulated genes 1
Cxcl12	NM_001033883	1.69	40	10858165	chemokine (C-X-C motif) ligand 12 (stromal cell-derived factor 1)
Ghr	NM_017094	1.28	15	10821632	growth hormone receptor
Pdgfrb	NM_031525	1.88	36	10802040	platelet derived growth factor receptor, beta polypeptide
**Immune Response**					

A2m	NM_012488	2.50	332	10858408	alpha-2-macroglobulin
C1qa	NM_001008515	1.63	14	10880734	complement component 1, q subcomponent, A chain
C7	AF309948	1.58	16	10821666	complement component 7
Cd97	NM_001012164	1.46	28	10806806	CD97 molecule
Ctage5	NM_001106734	1.21	69	10884772	CTAGE <Cutaneous T-cell lymphoma-associated antigen 5> family, membe
Fcgr2a	NM_053843	1.44	11	10769797	Fc fragment of IgG, low affinity IIa, receptor (CD32)
Fcgrt	NM_033351	1.60	84	10721667	Fc fragment of IgG, receptor, transporter, alpha
Ifi47	NM_172019	2.41	83	10733056	interferon gamma inducible protein 47
Irgm	NM_001012007	1.75	17	10742348	immunity-related GTPase family, M
RT1-A1	NM_001008827	1.27	21	10828417	RT1 class Ia, locus A1
RT1-CE15	NM_001008838	1.32	23	10833979	RT1 class I, locus CE15
RT1-DMa	NM_198741	1.26	21	10831628	RT1 class II, locus DMa
RT1-DMb	NM_198740	2.55	24	10831620	RT1 class II, locus DMb
Spa17	NM_053482	0.79	-35	10916232	sperm autoantigenic protein 17
Xkr8	NM_001012099	1.34	11	10872681	XK, Kell blood group complex subunit-related family, member 8
**Metabolism & Transport**					
Aadat	NM_017193	2.69	103	10791478	aminoadipate aminotransferase
Abhd14b	NM_001007664	1.26	108	10912845	abhydrolase domain containing 14b
Abhd4	NM_001108866	1.57	505	10780179	abhydrolase domain containing 4
Abhd6	NM_001007680	1.26	53	10782695	abhydrolase domain containing 6
Acox1	NM_017340	1.30	62	10749172	acyl-Coenzyme A oxidase 1, palmitoyl
Acsl1	NM_012820	1.52	28	10791677	acyl-CoA synthetase long-chain family member 1
Acsl5	NM_053607	1.21	33	10716168	acyl-CoA synthetase long-chain family member 5
Agpat2	NM_001107821	1.51	238	10843838	1-acylglycerol-3-phosphate O-acyltransferase 2 (lysophosphatidic acid acyl
Akr7a2	NM_134407	1.23	40	10873378	aldo-keto reductase family 7, member A2 (aflatoxin aldehyde reductase)
Aldh1a1	NM_022407	1.56	710	10714323	aldehyde dehydrogenase 1 family, member A1
Aldh3b1	NM_001006998	1.66	48	10727405	aldehyde dehydrogenase 3 family, member B1
Apobec3f	NM_001033703	1.24	13	10897723	apolipoprotein B mRNA editing enzyme, catalytic polypeptide-like 3F
Aprt	NM_001013061	1.27	27	10811651	adenine phosphoribosyl transferase
Bcat2	NM_022400	1.33	50	10706884	branched chain aminotransferase 2, mitochondrial
Bckdha	NM_012782	1.30	89	10719863	branched chain ketoacid dehydrogenase E1, alpha polypeptide
Bco2	NM_001127712	1.35	38	10917215	beta-carotene oxygenase 2
Clic2	NM_001009651	2.32	31	10827592	chloride intracellular channel 2
Cpt1a	NM_031559	1.45	158	10712657	carnitine palmitoyltransferase 1a, liver
Ddt	NM_024131	1.22	223	10832551	D-dopachrome tautomerase
Dhcr24	NM_001080148	1.51	146	10870567	24-dehydrocholesterol reductase
Dhcr7	NM_022389	1.33	362	10712560	7-dehydrocholesterol reductase
Echs1	NM_078623	1.24	149	10726611	enoyl Coenzyme A hydratase, short chain, 1, mitochondrial
Exosc5	NM_001107493	1.30	57	10705202	exosome component 5
Exosc6	ENSRNOT0000002	1.32	17	10808063	exosome component 6
Fads1	NM_053445	1.57	352	10713857	fatty acid desaturase 1
Fads2	NM_031344	1.36	316	10728631	fatty acid desaturase 2
Fads3	NM_173137	1.34	18	10713844	fatty acid desaturase 3
Fads6	NM_001107064	1.51	88	10748922	fatty acid desaturase domain family, member 6
Fmo1	NM_012792	2.26	42	10769361	flavin containing monooxygenase 1
Fuca2	NM_001004218	1.44	74	10701817	fucosidase, alpha-L- 2, plasma
Gcat	NM_001024277	1.59	16	10897574	glycine C-acetyltransferase (2-amino-3-ketobutyrate-coenzyme A ligase)
Gltpd1	NM_001007703	1.28	15	10882234	glycolipid transfer protein domain containing 1
Got1	NM_012571	1.60	365	10715364	glutamic-oxaloacetic transaminase 1, soluble (aspartate aminotransferase
Got2	NM_013177	1.26	87	10805794	glutamic-oxaloacetic transaminase 2, mitochondrial (aspartate aminotran
Gsta3	NM_031509	1.95	1197	10926958	glutathione S-transferase A3
Gstt1	NM_053293	1.71	127	10832555	glutathione S-transferase theta 1

Gstt2	NM_012796	1.21	64	10829653	glutathione S-transferase, theta 2
Gstt3	NM_001137643	1.72	151	10832563	glutathione S-transferase, theta 3
Hbb	NM_033234	0.46	-1078	10724315	hemoglobin, beta
Hibadh	NM_022243	1.37	241	10862586	3-hydroxyisobutyrate dehydrogenase
Hmgcr	NM_013134	1.43	335	10820666	3-hydroxy-3-methylglutaryl-Coenzyme A reductase
Hpx	NM_053318	2.28	72	10724483	hemopexin
Idh1	NM_031510	1.35	780	10928563	isocitrate dehydrogenase 1 (NADP+), soluble
Isoc1	NM_001014242	1.21	110	10801929	isochorismatase domain containing 1
Ivd	NM_012592	1.21	67	10838704	isovaleryl coenzyme A dehydrogenase
Lactb	NM_001106833	1.32	23	10918385	lactamase, beta
Lcat	NM_017024	0.78	-14	10810736	lecithin cholesterol acyltransferase
Lipe	NM_012859	1.44	26	10719829	lipase, hormone sensitive
LOC308670	ENSRNOT0000001	0.70	-16	10707572	pink-eyed dilution
Lpl	NM_012598	1.37	10	10791250	lipoprotein lipase
Me1	NM_012600	1.35	871	10919103	malic enzyme 1, NADP(+)-dependent, cytosolic
Mfsd1	XM_342258	1.31	50	10815850	major facilitator superfamily domain containing 1
Mgst1	NM_134349	1.44	329	10859392	microsomal glutathione S-transferase 1
Mvd	NM_031062	1.83	104	10811560	mevalonate (diphospho) decarboxylase
Nadk	NM_001109678	1.24	89	10874655	NAD kinase
Nampt	NM_177928	1.24	35	10884162	nicotinamide phosphoribosyltransferase
Nit1	NM_182668	1.37	121	10769870	nitrilase 1
Nsdhl	NM_001009399	1.43	99	10935845	NAD(P) dependent steroid dehydrogenase-like
Pafah2	NM_177932	1.29	23	10872790	platelet-activating factor acetylhydrolase 2
Pctk3	NM_001100506	1.21	11	10767577	PCTAIRE protein kinase 3
Pcyox1l	NM_001134542	1.30	46	10804714	prenylcysteine oxidase 1 like
Piga	NM_001108816	0.81	-22	10937719	phosphatidylinositol glycan anchor biosynthesis, class A
Plb1	NM_138898	1.93	31	10888701	phospholipase B1
Pnpla2	NM_001108509	1.38	80	10712317	patatin-like phospholipase domain containing 2
Pold4	NM_001013195	1.37	16	10712858	polymerase (DNA-directed), delta 4
Ppa2	NM_001135871	1.21	27	10819116	pyrophosphatase (inorganic) 2
Ppap2a	NM_022538	1.65	79	10813048	phosphatidic acid phosphatase type 2A
Prr13	NM_001008379	1.23	133	10899566	proline rich 13
Psap	NM_013013	1.35	858	10829976	prosaposin
Pyroxd2	NM_001004261	1.27	40	10715400	pyridine nucleotide-disulphide oxidoreductase domain 2
Qsox2	NM_001109434	1.28	159	10843664	quiescin Q6 sulfhydryl oxidase 2
RGD1303003	NM_001004225	1.29	49	10829265	homolog of zebrafish ES1
RGD1562373	NM_001040019	1.26	20.39	10920820	similar to 3-ketoacyl-CoA thiolase B, peroxisomal precursor (Beta-ketothio
Rhbdf1	NM_001030034	1.20	11	10732607	rhomboid 5 homolog 1 (Drosophila)
Sc4mol	NM_080886	1.83	1163	10787841	sterol-C4-methyl oxidase-like
Sc5dl	NM_053642	1.59	155	10916534	sterol-C5-desaturase (ERG3 delta-5-desaturase homolog, S. cerevisiae)-lik
Scd	NM_031841	1.24	848	10715546	stearoyl-CoA desaturase (delta-9-desaturase)
Sclt1	NM_153740	0.77	-15	10822989	sodium channel and clathrin linker 1
Scrn3	NM_001013162	0.82	-28	10836973	secernin 3
Selenbp1	NM_080892	1.58	72	10824892	selenium binding protein 1
Sfxn1	NM_001012213	1.70	70	10797475	sideroflexin 1
Sh3bgrl3	NM_001106688	1.46	76	10880436	SH3 domain binding glutamic acid-rich protein-like 3
Slc1a3	NM_019225	1.54	11	10821824	solute carrier family 1 (glial high affinity glutamate transporter), member
Slc25a1	NM_017307	1.30	125	10752367	solute carrier family 25 (mitochondrial carrier, citrate transporter), memb
Slc25a40	NM_001037186	0.80	-18	10853292	solute carrier family 25, member 40
Slc27a1	NM_053580	1.21	134	10787313	solute carrier family 27 (fatty acid transporter), member 1
Slc27a3	NM_001106439	1.38	40	10824611	solute carrier family 27 (fatty acid transporter), member 3
Slc36a1	NM_130415	1.25	47	10733697	solute carrier family 36 (proton/amino acid symporter), member 1

Slc39a6	NM_001024745	1.21	73	10803520	solute carrier family 39 (zinc transporter), member 6
Slc7a4	NM_001107078	1.38	35	10752433	solute carrier family 7 (cationic amino acid transporter, y+ system), memb
Slc9a3r1	NM_021594	1.52	54	10739490	solute carrier family 9 (sodium/hydrogen exchanger), member 3 regulator
Smpd1	NM_001006997	1.25	188	10709575	sphingomyelin phosphodiesterase 1, acid lysosomal
Sod2	NM_017051	1.35	446	10717935	superoxide dismutase 2, mitochondrial
Sord	NM_017052	1.33	195	10839254	sorbitol dehydrogenase
Sphk1	NM_133386	1.30	10	10739796	sphingosine kinase 1
Sptlc1	NM_001108406	1.33	90	10794206	serine palmitoyltransferase, long chain base subunit 1
Sqle	NM_017136	1.58	194	10896772	squalene epoxidase
Star	NM_031558	1.64	1240	10792251	steroidogenic acute regulatory protein
Stard4	NM_001106159	1.31	21	10803681	StAR-related lipid transfer (START) domain containing 4
Sumf1	NM_001108639	1.28	31	10864540	sulfatase modifying factor 1
Tap1	NM_032055	1.44	28	10831606	transporter 1, ATP-binding cassette, sub-family B (MDR/TAP)
Trappc6a	NM_001109410	1.39	33	10704849	trafficking protein particle complex 6A
Tst	NM_012808	1.82	242	10905284	thiosulfate sulfurtransferase
Unc93b1	NM_001108513	1.58	83	10712720	unc-93 homolog B1 (C. elegans)
Vat1	NM_001033683	1.35	64	10747633	vesicle amine transport protein 1 homolog (T californica)
Pgd	ENSRNOT0000001	1.23	182	10881671	phosphogluconate dehydrogenase
**Proteolysis**					
Ctsd	NM_134334	1.48	564	10726979	cathepsin D
Ctsf	NM_001034110	1.36	40	10712935	cathepsin F
Hecw1	NM_001106117	0.69	-37	10795482	HECT, C2 and WW domain containing E3 ubiquitin protein ligase 1
Mmp2	NM_031054	1.58	52	10809540	matrix metallopeptidase 2
Mon1b	NM_001107433	1.49	51	10808138	MON1 homolog b (yeast)
Pcolce	NM_019237	1.51	405	10760971	procollagen C-endopeptidase enhancer
Pkia	NM_053772	1.26	35	10822386	protein kinase (cAMP-dependent, catalytic) inhibitor alpha
Psmb10	NM_001025637	1.24	14	10810727	proteasome (prosome, macropain) subunit, beta type 10
Psmb8	NM_080767	1.72	41	10831595	proteasome (prosome, macropain) subunit, beta type 8 (large multifunct
Spinlw1	NM_001109457	0.74	-139	10851628	serine peptidase inhibitor-like, with Kunitz and WAP domains 1 (eppin)
Timp1	NM_053819	1.51	474	10936482	TIMP metallopeptidase inhibitor 1
Ube2n	BC090072	0.73	-12	10759435	ubiquitin-conjugating enzyme E2N
Vps28	NM_001130492	1.27	144	10905040	vacuolar protein sorting 28 homolog (S. cerevisiae)
Wfdc10	NM_001109461	0.69	-121	10842156	WAP four-disulfide core domain 10
Xpnpep2	NM_057155	2.10	84	10935289	X-prolyl aminopeptidase (aminopeptidase P) 2, membrane-bound
**Receptors & Binding Proteins**					
Abcc4	NM_133411	1.40	79	10785846	ATP-binding cassette, sub-family C (CFTR/MRP), member 4
Chp	NM_024139	1.37	157	10876827	calcium binding protein p22
Chrnb4	NM_052806	1.40	22	10917607	cholinergic receptor, nicotinic, beta 4
Dbp	NM_012543	1.46	49	10706953	D site of albumin promoter (albumin D-box) binding protein
Ebp	NM_057137	1.62	81	10932410	emopamil binding protein (sterol isomerase)
Ebpl	NM_001108381	1.36	13	10784355	emopamil binding protein-like
F2r	NM_012950	1.57	73	10820586	coagulation factor II (thrombin) receptor
Leng4	NM_001134978	1.31	60	10703715	leukocyte receptor cluster (LRC) member 4
LOC683722	NM_001101008	0.62	-27	10718111	similar to Fgfr1 oncogene partner
Lrp10	NM_001037777	1.33	137	10780218	low-density lipoprotein receptor-related protein 10
Lrp11	NM_001106217	1.23	15	10701699	low density lipoprotein receptor-related protein 11
Mrap	NM_001135834	1.52	193	10750173	melanocortin 2 receptor accessory protein
Npffr1	NM_022291	0.55	-52	10830034	neuropeptide FF receptor 1
Pepd	NM_001009641	1.26	151	10706117	peptidase D
Pvrl2	NM_001012064	1.39	33	10719549	poliovirus receptor-related 2
Renbp	NM_031095	1.23	14	10940260	renin binding protein
Sigmar1	NM_030996	1.22	60	10876185	sigma non-opioid intracellular receptor 1

Tax1bp3	NM_001025419	1.36	118	10735662	Tax1 (human T-cell leukemia virus type I) binding protein 3
Tm7sf2	NM_001013071	2.26	195	10728123	transmembrane 7 superfamily member 2
Tmem110	NM_198774	1.22	24	10786522	transmembrane protein 110
Tmem126b	NM_001106280	1.26	34	10723644	transmembrane protein 126B
Tmem147	NM_001038494	1.31	93	10720767	transmembrane protein 147
Tmem150c	NM_001108354	1.80	53	10771456	transmembrane protein 150C
Tmem19	NM_199098	1.21	30	10902401	transmembrane protein 19
Tmem20	XM_220029	1.66	158	10715149	transmembrane protein 20
Tmem205	NM_001106804	1.53	81	10915657	transmembrane protein 205
Tmem62	ENSRNOT0000003	1.23	19	10839075	transmembrane protein 62
Tmem8	NM_001106991	1.27	20.23	10732534	transmembrane protein 8 (five membrane-spanning domains)
Tmem86a	NM_001135016	1.23	687	10707151	transmembrane protein 86A
**Signaling**					
Acvrl1	NM_022441	1.41	11	10899354	activin A receptor type II-like 1
Agfg2	NM_001107131	1.25	15	10760994	ArfGAP with FG repeats 2
Ak3	NM_013218	1.21	103	10729590	adenylate kinase 3
Anxa11	NM_001011918	1.28	67	10786174	annexin A11
Anxa6	NM_024156	1.31	232	10742712	annexin A6
Apln	NM_031612	1.77	27	10939564	apelin
Arhgap12	NM_001107357	0.70	-177	10795574	Rho GTPase activating protein 12
Aurkaip1	NM_001004237	1.23	100	10874723	aurora kinase A interacting protein 1
Avpi1	NM_134373	2.11	195	10715452	arginine vasopressin-induced 1
Bambi	NM_139082	0.75	-117	10798850	BMP and activin membrane-bound inhibitor, homolog (Xenopus laevis)
Btbd12	ENSRNOT0000003	0.81	-22	10731872	BTB (POZ) domain containing 12
Cib1	NM_031145	1.41	83	10723032	calcium and integrin binding 1 (calmyrin)
Crkrs	NM_001033867	0.80	-42	10737927	Cdc2-related kinase, arginine/serine-rich
Dclk2	NM_001009691	1.44	23	10824091	doublecortin-like kinase 2
Dlc1	NM_001127446	1.35	22	10788462	deleted in liver cancer 1
Dtx3l	NM_001109053	1.44	48	10751459	deltex 3-like (Drosophila)
Dusp16	NM_001106624	1.39	51	10866401	dual specificity phosphatase 16
Eepd1	NM_001014088	1.44	35	10908682	endonuclease/exonuclease/phosphatase family domain containing 1
Egfl7	NM_139104	1.39	11	10834447	EGF-like-domain, multiple 7
Ehd2	NM_001024897	1.58	32	10719204	EH-domain containing 2
Farp2	NM_001108233	1.30	17	10925609	FERM, RhoGEF and pleckstrin domain protein 2
Fastkd5	XR_086207	1.28	27	10849925	FAST kinase domains 5
Fert2	NM_001106928	0.78	-30	10925761	fer (fms/fps related) protein kinase, testis specific 2
Fkbp1a	NM_013102	1.28	62	10840741	FK506 binding protein 1a
Frmd8	NM_001008348	1.32	32	10728030	FERM domain containing 8
Fxyd5	NM_021909	2.23	48	10720859	FXYD domain-containing ion transport regulator 5
Gm2a	NM_172335	1.47	441	10733690	GM2 ganglioside activator
Gpr176	ENSRNOT0000000	1.82	26	10848429	G protein-coupled receptor 176
Gpr177	NM_001085353	1.23	234	10819975	G protein-coupled receptor 177
Impa2	NM_172224	0.71	-10	10802407	inositol (myo)-1(or 4)-monophosphatase 2
Insig1	NM_022392	1.47	480	10861986	insulin induced gene 1
Khdrbs3	NM_022249	1.22	16	10896948	KH domain containing, RNA binding, signal transduction associated 3
Magmas	NM_001100136	1.59	24	10731783	mitochondria-associated protein involved in granulocyte-macrophage
Mapk13	NM_019231	1.43	11	10828778	mitogen activated protein kinase 13
Mobkl2a	NM_001108734	1.34	31	10893739	MOB1, Mps One Binder kinase activator-like 2A (yeast)
Nxn	NM_001108285	1.53	78	10736545	nucleoredoxin
P2ry14	NM_133577	1.46	14	10823353	purinergic receptor P2Y, G-protein coupled, 14
Pex6	NM_057125	1.27	34	10921677	peroxisomal biogenesis factor 6
Phlda3	NM_001012206	1.54	13	10764228	pleckstrin homology-like domain, family A, member 3

Plekhb2	NM_001106899	1.30	93	10922476	pleckstrin homology domain containing, family B (evectins) member 2
Plekhm1	NM_001009677	1.24	24	10748061	pleckstrin homology domain containing, family M (with RUN domain)
Prkcdbp	NM_134449	1.59	57	10724464	protein kinase C, delta binding protein
Rab30	NM_001015012	0.78	-15	10708665	RAB30, member RAS oncogene family
Rab31	NM_145094	1.72	58	10930259	RAB31, member RAS oncogene family
Rab33a	NM_001108257	2.12	64	10935353	RAB33A, member of RAS oncogene family
Rab5c	NM_001105840	1.22	111	10747459	RAB5C, member RAS oncogene family
Rap1a	NM_001005765	0.75	-84	10825727	RAP1A, member of RAS oncogene family
RGD1562220	ENSRNOT0000000	0.61	-322	10771492	similar to GPI-gamma 4; GPIgamma4
RGD1562230	BC166848	0.72	-25	10829933	similar to catenin alpha 3
RGD1563970	ENSRNOT0000005	1.76	22	10724391	similar to Tripartite motif protein 30-like
RGD1564174	AB510354	1.72	137	10778404	similar to novel protein similar to Tensin Tns
Rhoj	NM_001008320	1.81	41	10885299	ras homolog gene family, member J
Ssu72	NM_001025657	1.29	181	10874693	SSU72 RNA polymerase II CTD phosphatase homolog (S. cerevisiae)
Stk19	NM_001013197	1.20	13	10828221	serine/threonine kinase 19
Tbc1d25	NM_001106955	1.32	21	10932416	TBC1 domain family, member 25
Tns4	NM_001024881	1.38	21	10747011	tensin 4
Vrk2	NM_001108366	0.75	-16	10778806	vaccinia related kinase 2
Ywhab	NM_019377	1.21	236	10842052	tyrosine 3-monooxygenase/tryptophan 5-monooxygenase activation
**Transcription**					
Ankrd26	ENSRNOT0000005	0.76	-13	10864979	ankyrin repeat domain 26
Asb3	NM_001108864	0.78	-38	10774880	ankyrin repeat and SOCS box-containing 3
Atf5	NM_172336	1.43	20.01	10721480	activating transcription factor 5
Casc5	ENSRNOT0000006	0.47	-94	10838741	cancer susceptibility candidate 5
Ccdc112	NM_001109124	0.75	-26	10804371	coiled-coil domain containing 112
Ccdc163	NM_001025656	0.63	-14	10871293	coiled-coil domain containing 163
Ccdc34	NM_001108587	0.75	-25	10838373	coiled-coil domain containing 34
Ccdc46	NM_001105849	0.60	-24	10739282	coiled-coil domain containing 46
Ccdc55	NM_001037189	0.72	-37	10736476	coiled-coil domain containing 55
Ccdc62	NM_001134766	0.75	-13	10761792	coiled-coil domain containing 62
Dnajc15	NM_001106050	1.48	153	10785372	DnaJ (Hsp40) homolog, subfamily C, member 15
Dnajc21	NM_138856	0.81	-19	10821900	DnaJ (Hsp40) homolog, subfamily C, member 21
Dnlz	NR_024073	1.28	79	10843693	DNL-type zinc finger
Eny2	NM_001130580	0.77	-132	10896400	enhancer of yellow 2 homolog (Drosophila)
Esr1	NM_012689	1.38	15	10702689	estrogen receptor 1
Esr2	NM_012754	0.79	-125	10890654	estrogen receptor 2 (ER beta)
Fbxo16	NM_001013132	1.31	15	10781089	F-box protein 16
Fbxo33	NM_001108023	1.23	19.51	10890156	F-box protein 33
Hes1	NM_024360	0.83	-69	10754943	hairy and enhancer of split 1 (Drosophila)
Ipo4	NM_001106038	1.27	56	10783818	importin 4
Klhdc8a	NM_001100683	1.90	173	10763883	kelch domain containing 8A
Litaf	NM_001105735	1.35	31	10731493	lipopolysaccharide-induced TNF factor
Mdm4	NM_001012026	0.64	-141	10767663	Mdm4 p53 binding protein homolog (mouse)
Ndrg2	NM_133583	1.51	194	10783213	N-myc downstream regulated gene 2
Nrip1	NM_001100560	0.79	-187	10752754	nuclear receptor interacting protein 1
RGD1306820	NM_001107557	0.71	-25	10711743	similar to erythroid differentiation-related factor 1
Sarnp	NM_001033070	0.79	-162	10893247	SAP domain containing ribonucleoprotein
Scai	ENSRNOT0000003	0.63	-69	10844916	suppressor of cancer cell invasion
Sirt7	NM_001107073	1.32	50	10749704	sirtuin 7 (silent mating type information regulation 2, homolog) 7
Snrpf	NM_001126091	0.77	-38	10901920	small nuclear ribonucleoprotein polypeptide F
Srebf2	NM_001033694	1.21	82	10898091	sterol regulatory element binding transcription factor 2
Stat1	NM_032612	1.53	104	10927842	signal transducer and activator of transcription 1

Stat5b	NM_022380	1.58	91	10747494	signal transducer and activator of transcription 5B
Stat6	NM_001044250	1.40	27	10895932	signal transducer and activator of transcription 6
RGD1304792	NM_001134554	0.79	-13	10856502	similar to chromosome 2 open reading frame 3; transcription factor 9
Tcf20	NM_001130574	1.21	32	10905765	transcription factor 20
Zdhhc9	NM_001039016	1.21	15	10939570	zinc finger, DHHC-type containing 9
Zfp207	NM_001039020	0.82	-178	10736647	zinc finger protein 207
Zfp40	NM_001168642	0.72	-22	10718436	zinc finger protein 40
Znf184	NM_001100573	0.82	-25	10798479	zinc finger protein 184
**Translation & Protein Modification**					
Cpsf6	NM_001106785	0.77	-70	10902552	cleavage and polyadenylation specific factor 6
Ddx54	ENSRNOT0000000	1.35	38	10762275	DEAD (Asp-Glu-Ala-Asp) box polypeptide 54
Fusip1	NM_001025738	0.82	-64	10872929	FUS interacting protein (serine-arginine rich) 1
Gars	ENSRNOT0000001	1.21	189	10862666	glycyl-tRNA synthetase
Gemin4	NM_001109037	1.21	23	10745022	gem (nuclear organelle) associated protein 4
Hnrnpa1	NM_017248	0.83	-417	10932740	heterogeneous nuclear ribonucleoprotein A1
Hnrnpa2b1	NM_001104613	0.80	-322	10860773	heterogeneous nuclear ribonucleoprotein A2/B1
Hnrnpa3	NM_001111295	0.81	-401	10823933	heterogeneous nuclear ribonucleoprotein A3
Iars	NM_001100572	1.41	48	10797597	isoleucyl-tRNA synthetase
Mrpl36	NM_001108879	1.29	63	10796835	mitochondrial ribosomal protein L36
Mrps18a	NM_198756	1.22	56	10926570	mitochondrial ribosomal protein S18A
Ncl	NM_012749	0.80	-106	10779091	nucleolin
Npm1	NM_012992	0.79	-427	10893361	nucleophosmin (nucleolar phosphoprotein B23, numatrin)
Padi2	NM_017226	1.60	17	10873580	peptidyl arginine deiminase, type II
Paip2l1	ENSRNOT0000002	0.75	-72	10710769	polyadenylate-binding protein-interacting protein 2-like 1
Parp12	NM_001108623	1.21	24	10862014	poly (ADP-ribose) polymerase family, member 12
Ptrh2	NM_001013860	1.30	42	10737125	peptidyl-tRNA hydrolase 2
Pwp2	NM_001168653	1.39	49	10829244	PWP2 periodic tryptophan protein homolog (yeast)
Qk	NM_001115021	0.81	-65	10703111	quaking
Rbm43	NM_001037649	0.79	-21	10845130	RNA binding motif protein 43
Rbm47	NM_001005882	1.72	152	10772657	RNA binding motif protein 47
Rpl7a	NM_001114391	0.75	-21	10797321	ribosomal protein L7a
Rpsa	NM_017138	1.20	268	10914308	ribosomal protein SA
Scap	NM_001100966	1.44	126	10913640	SREBF chaperone
Slfn2	NM_001107031	1.95	69	10736795	schlafen 2
Syncrip	NM_001047916	0.79	-112	10919214	synaptotagmin binding, cytoplasmic RNA interacting protein
Tbce	NM_001012161	0.81	-59	10795528	tubulin folding cofactor E
Tia1	NM_001012096	0.68	-142	10856971	TIA1 cytotoxic granule-associated RNA binding protein
Tra2a	NM_001126296	0.76	-166	10862444	transformer 2 alpha homolog (Drosophila)
Yars	NM_001025696	1.24	42	10872291	tyrosyl-tRNA synthetase
**Miscellaneous & Unknown**					
Bsdc1	NM_001106636	1.22	56	10872326	BSD domain containing 1
Fam108a1	NM_001006983	1.26	38	10893768	family with sequence similarity 108, member A1
Fam122b	NM_001166586	0.60	-37	10939805	family with sequence similarity 122B
Fam126a	ENSRNOT0000001	1.39	172	10852953	family with sequence similarity 126, member A
Fam49b	BC166469	0.81	-228	10904018	family with sequence similarity 49, member B
Fam73b	NM_001106566	1.29	35	10835213	family with sequence similarity 73, member B
LOC500420	BC089864	1.21	28	10875751	similar to CG12279-PA
LOC500625	NM_001025151	0.69	-52	10883423	hypothetical protein LOC500625
Morn4	NM_001024975	1.20	23	10715455	MORN repeat containing 4
RGD1305689	NM_001008297	1.44	289	10789740	similar to DNA segment, Chr 14, ERATO Doi 449, expressed
RGD1307882	ENSRNOT0000001	0.73	-12	10853900	similar to CG9346-PA
RGD1308616	XM_001069457	0.78	-12	10879217	similar to KIAA0467 protein

RGD1309228	NM_001017451	1.38	76	10889027	similar to putative protein, with at least 9 transmembrane domains,
RGD1310769	NM_001106749	1.30	118	10891297	similar to HSPC288
RGD1311422	ENSRNOT0000000	1.40	23	10748935	similar to CG8841-PA
RGD1559961	NM_001163736	0.83	-24	10745785	similar to novel protein
Romo1	ENSRNOT0000002	1.22	39	10841484	reactive oxygen species modulator 1
Setd3	ENSRNOT0000000	1.29	48	10892118	SET domain containing 3
Spryd4	NM_001037765	1.46	38	10899736	SPRY domain containing 4
Wdr45	NM_001013958	1.22	51	10937064	WD repeat domain 45
Wdr46	NM_212491	1.34	32	10831747	WD repeat domain 46
Wdr6	NM_001006988	1.26	60	10920313	WD repeat domain 6
Wdr90	XM_213271	0.73	-14	10741606	WD repeat domain 90
**EST's**					
RGD1307704	NM_001108040	1.35	27	10885849	similar to RIKEN cDNA 2410016O06
RGD1309906	NM_001009246	1.26	47	10743723	similar to RIKEN cDNA 2310004I24 gene
RGD1309926	ENSRNOT0000000	1.55	18	10732657	similar to RIKEN cDNA G431001E03 gene
RGD1560207	XM_576972	0.54	-18	10938176	similar to chromosome X open reading frame 22
RGD1560277	XM_001066043	1.46	23	10708167	similar to RIKEN cDNA D330012F22 gene
RGD1563680	ENSRNOT0000000	0.80	-17	10838559	similar to CDNA sequence BC052040
	ENSRNOT0000005	0.66	-383	10788858	
	---	0.74	-254	10862453	
	---	0.64	-248	10700398	
	ENSRNOT0000005	0.69	-213	10772758	
	---	0.53	-207	10714204	
	---	0.82	-193	10700124	
	ENSRNOT0000003	0.80	-154	10903977	
	ENSRNOT0000004	0.77	-144	10797009	
	ENSRNOT0000006	0.78	-129	10793597	
	---	0.66	-113	10700714	
	---	0.65	-110	10701207	
	---	0.77	-110	10700841	
	GENSCAN0000002	0.78	-102	10919226	
	ENSRNOT0000005	0.57	-75	10713600	
	---	0.56	-72	10701246	
	ENSRNOT0000004	0.79	-72	10719074	
	ENSRNOT0000004	0.79	-72	10746652	
	---	0.74	-63	10701044	
	ENSRNOT0000004	0.65	-63	10738162	
	---	0.47	-56	10700268	
	---	0.73	-55	10765036	
	---	0.82	-53	10701109	
	ENSRNOT0000005	0.71	-51	10834598	
	ENSRNOT0000005	0.70	-48	10802710	
	ENSRNOT0000003	0.82	-47	10825062	
	AJ240056	0.75	-47	10733045	
	AJ240056	0.77	-46	10733047	
	---	0.51	-44	10700624	
	---	0.42	-43	10700213	
	---	0.56	-42	10700311	
	ENSRNOT0000005	0.69	-39	10910768	
	---	0.47	-39	10700112	
	---	0.54	-38	10701019	
	---	0.58	-35	10700580	

	---	0.78	-33	10926095	
	ENSRNOT0000005	0.71	-31	10736238	
	---	0.74	-31	10700068	
	ENSRNOT0000003	0.79	-31	10809766	
	ENSRNOT0000005	0.70	-31	10832840	
	ENSRNOT0000005	0.70	-30	10802706	
	ENSRNOT0000005	0.67	-28	10802708	
	ENSRNOT0000005	0.60	-26	10864356	
	AY539927	0.53	-24	10868302	
	ENSRNOT0000000	0.83	-24	10767299	
	ENSRNOT0000000	0.75	-23	10898947	
	rno-mir-16	0.58	-23	10815915	
	ENSRNOT0000005	0.72	-22	10887336	
	ENSRNOT0000005	0.75	-22	10797019	
	ENSRNOT0000006	0.76	-21	10805514	
	---	0.70	-19.8	10815943	
	rno-mir-7a-1	0.75	-19	10793838	
	ENSRNOT0000005	0.75	-19	10713606	
	---	0.71	-18	10708587	
	---	0.61	-18	10701547	
	---	0.54	-17	10700141	
	---	0.81	-17	10700303	
	---	0.73	-14	10701560	
	GENSCAN0000001	0.77	-14	10901103	
	---	0.60	-13	10700855	
	ENSRNOT0000004	0.80	-13	10809266	
	ENSRNOT0000005	0.64	-12	10878963	
	---	0.80	-12	10700840	
	---	0.54	-12	10700934	
	---	0.81	-12	10908131	
	ENSRNOT0000005	0.73	-12	10756268	
	ENSRNOT0000005	0.73	-12	10756270	
	ENSRNOT0000005	0.73	-12	10756272	
	ENSRNOT0000005	0.73	-12	10759445	
	---	0.72	-11	10700214	
	---	0.56	-10	10700520	
	ENSRNOT0000003	0.75	-10	10818946	
	ENSRNOT0000005	0.70	-10	10888682	
	---	1.56	10	10700584	
	ENSRNOT0000006	1.27	11	10863606	
	ENSRNOT0000000	1.36	11	10904864	
	BC089062	1.41	12	10877753	
	---	1.33	12	10752863	
	ENSRNOT0000006	1.29	12	10750810	
	ENSRNOT0000005	1.32	12	10793367	
	ENSRNOT0000005	1.32	16	10798255	
	ENSRNOT0000005	1.37	17	10722449	
	---	1.36	18	10726672	
	---	1.43	19	10700788	
	---	1.52	21	10700608	
	ENSRNOT0000006	1.39	22	10913991	
	---	1.22	23	10700247	

	ENSRNOT0000005	1.25	23	10820280	
	---	1.46	25	10700882	
	ENSRNOT0000004	1.34	27	10766057	
	---	1.48	28	10701440	
	---	1.52	35	10866408	
	ENSRNOT0000004	1.25	36	10854091	
	ENSRNOT0000002	1.23	42	10798900	
	ENSRNOT0000002	1.23	44	10880530	
	---	1.40	44	10700996	
	---	1.49	45	10701505	
	---	1.49	54	10700409	
	ENSRNOT0000005	1.28	55	10770543	
	---	1.66	56	10700706	
	---	1.62	65	10701568	
	---	1.25	68	10700922	
	ENSRNOT0000001	1.27	80	10865483	
	---	2.03	93	10700752	
	---	1.58	100	10700861	
	---	1.55	104	10700365	
	---	1.22	108	10701172	
	---	1.20	114	10701058	
	ENSRNOT0000000	1.52	119	10751469	
	---	1.30	130	10700223	
	---	1.41	157	10700881	
	---	1.22	168	10701153	
	---	1.36	231	10701066	
	J05014	1.31	1008	10876313	
