# Supplementary material for: Environmentally Induced Epigenetic Transgenerational Inheritance of Ovarian Disease
Source: PLoS One. 2012 May 3;7(5):e36129. doi: 10.1371/journal.pone.0036129 (PMC3343040; doi:10.1371/journal.pone.0036129)
Supplement: Table S2 — Differential expressed gene clusters. (PDF) [file pone.0036129.s004.pdf]

Supplemental Table S2

DIFFERENTIAL EXPRESSED GENE CLUSTERS

	CLUSTER LOCATION	DMR INSIDE OR WITHIN 2MB OF A CLUSTER	GENES WITH EXPRESSION CHANGE IN THE CLUSTER
**1**	chr1:78900000-81000000	Ceacam9 (within 2MB of cluster)	Pvrl2, Lipe, Bckdha, Exosc5
**2**	chr1:80250000-82650000		Lipe, Bckdha, Exosc5, Cyp2t1
**3**	chr1:85550000-87750000		Tmem147, Dmkn, Fxyd5, Pepd
**4**	chr1:94200000-97250000		Atf5, Fcgrt, Bcat2, Dbp
**5**	chr1:161300000-164150000		Hbb, RGD1563970, Prkcdbp, Smpd1, Hpx
**6**	chr1:204850000-208800000		Cpt1a, Aldh3b1, Unc93b1, Pold4, Ctsf, Frmd8, Tm7sf2
**7**	chr1:210700000-213350000		EST (chr1:211391510-211391584), EST (chr1:211392892-211392958), Fads1, Fads2, Fads3
**8**	chr1:246800000-250050000		Got1, Pyroxd2, Avpi1, Morn4, Scd
**9**	chr3:2800000-6450000	Lcn11 (inside cluster)	Npdc1, Qsox2, Dnlz, Egfl7, Agpat2, Rpl17a
**10**	chr5:58000000-61150000		EST (chr5:58166155-58168558), Sigmar1, Unc13b, NSCAN chr5.329.a (possibly RMRP), Tpm2
**11**	chr5:136900000-138950000		Hmgb1 (chr5:136955255-136955995), Ccdc163, RGD1308616, Rps8
**12**	chr5:151350000-154850000		Xkr8, Sh3bgrl3, Pafah2, NSCAN chr5.1149.a (possibly Sepn1), Fusip1
**13**	chr5:170750000-174300000		Nadk, Ssu72, Aurkaip1, Gltpd1
**14**	chr7:112700000-116000000		Scrib, NSCAN chr7.893.a (possibly Parp10) , Bop1, Vps28
**15**	chr10:13700000-16100000		Nubp2, Wdr90, Tmem8, Rhbdf1
**16**	chr10:32250000-36050000		Gnb2l1 (region 1), Gnb2l1 (region 2), Irgm, Ifi47
**17**	chr10:39100000-42350000	Vdac1 (within 2MB of cluster)	Anxa6, Gm2a, Slc36a1, Hmgb1 (chr10:41063919-41064554)
**18**	chr10:62500000-65550000		Gemin4, LOC360570, Pigs, Rpl23a
**19**	chr10:86850000-90800000	Plekhm1 (within 2MB of cluster)	Crkrs, Tns4, Krtap4-5, NSCAN chr10.1275.a, Rab5c, Stat5b, Vat1
**20**	chr10:103400000-107350000		Ttyh2, Slc9a3r1, Fdxr, Fads6, RGD1311422, Hmgb1 (chr10:105805282-105805930), Fbf1, Acox1, Sphk1
**21**	chr13:85400000-88800000		Fcgr2a (region 1), Fcgr2a (region 2), Nit1, F11r
**22**	chr15:31800000-34250000		Abhd4, Lrp10, Jub, Ipo4
**23**	chr17:6700000-8700000		RGD1561537, NSCAN chr17.055.a (region 1), NSCAN chr17.055.a (region 2), NSCAN chr17.056.a
**24**	chr20:2850000-6800000	Agpat1 (inside cluster)	Stk19, Psmb8, Tap1, RT1-DMb, RT1-Dma, RT1-A1, Wdr46
**25**	chr20:11650000-15150000	LOC686922 (inside cluster)	Col6a1, Gstt2, Ddt, Gstt1, Gstt3
