# Supplementary material for: Environmentally Induced Epigenetic Transgenerational Inheritance of Ovarian Disease
Source: PLoS One. 2012 May 3;7(5):e36129. doi: 10.1371/journal.pone.0036129 (PMC3343040; doi:10.1371/journal.pone.0036129)
Supplement: Table S3 — Doses and sources of chemicals used. (PDF) [file pone.0036129.s005.pdf]

Supplemental Table S3. Doses and Sources of Chemicals Used

Treatment ID	Chemical	Dose	Source	% Oral LD50 Dose
**Control**	Control (DSMO 50%)	100 µl/kg BW/d	Sigma Aldrich Corp, St. Louis, MO	
**Vinclozolin**	Vinclozolin	100 mg/kg BW/d	Chem Service, West Chester, PA	1%
**Pesticides**	Permethrin	150 mg/kg BW/d	Sigma Aldrich Corp, St. Louis, MO	39%
	DEET	40 mg/kg BW/d	Chem Service, West Chester, PA	2%
**Dioxin**	TCCD	100 ng/kg BW/d	Cambridge Isotope Labs, Andover, MA	0.1%
**Plastics**	Bisphenol A	50 mg/kg BW/d	Sigma Aldrich Corp, St. Louis, MO	1%
	DEHP	750 mg/kg BW/d	Sigma Aldrich Corp, St. Louis, MO	0.025%
	DBP	66 mg/kg BW/d	Sigma Aldrich Corp, St. Louis, MO	0.8%
**Jet Fuel**	JP8	500 mg/kg BW/d	Lt Dean Wagner, Dayton, OH	25%

The **low dose plastics** used 50% the dose listed above for BPA, DEHP and DBP
